# Reaction of NHOs with Bisphosphanes – Designing Diradicaloids, Zwitterions and Radicals

**DOI:** 10.1002/anie.202423347

**Published:** 2025-02-13

**Authors:** Karsten Paul Lüdtke, Edgar Zander, Florian Taube, Jan‐Erik Siewert, Björn Corzilius, Christian Hering‐Junghans, Jonas Bresien, Axel Schulz

**Affiliations:** ^1^ Anorganische Chemie Institut für Chemie Universität Rostock A.-Einstein-Str. 3a 18059 Rostock; ^2^ Leibniz Institut für Katalyse e. V. Albert-Einstein-Str. 29a 18059 Rostock; ^3^ Universität Rostock Department Light-Life-Mater Albert-Einstein-Straße 3a 18059 Rostock Germany

**Keywords:** phosphorus, diradical, molecule activation, *N*-heterocyclic olefin, zwitterion

## Abstract

The linkage of an imidazole‐based *N*‐heterocyclic olefin (NHO), containing a terminal CH_2_ donor group, with a phosphorus‐centered diradical molecular fragment leads to an open‐shell singlet diphospha‐indenylide system, a new class of *P*‐heterocycles, which can be interpreted both as a phosphorus‐centered diradicaloid and as a zwitterion with a permanent, overall charge separation between the *N*‐ and *P*‐heterocyclic ring systems. The rotation of the imidazole ring, which is thermally possible due to a central C−C bond with a weakened π‐component, changes both the charge separation and diradical character depending on the dihedral angle, as quantum mechanical calculations indicate. By varying the bulkiness of substituents at the imidazole‐based NHO, it was possible to obtain different diphospha‐indenylide species with different rotation angles in the solid state and hence varying diradical character. Imidazolium‐diphospha‐indenylides represent a new class of NHO‐based zwitterions with diradical character. Their synthesis, structure, and activation chemistry are described, as well as the quantum mechanical description of the electronic structure in these unusual heterocycles. In addition, along the synthesis route to diphospha‐indenylide, we also succeeded in isolating a highly reactive monoradical anion, which was also fully characterized.

## Graphical abstract

By linking *N*‐heterocyclic olefins with a P‐substituted indenyl heterocycle, it was possible to synthesize a very exciting new heterocyclic substance class that is both zwitterionic and diradical. The diradical character of these heterocycles can be adjusted by rotation within the molecule, which was achieved by rational design. The thermochromism of these heterocycles was studied by experimental and theoretical methods. Furthermore, we have shown that these heterocycles can activate small molecules. The mechanism of such an activation chemistry was studied and shown to be reversible in some case. Finally, we studied the electronic structure using multireference methods.

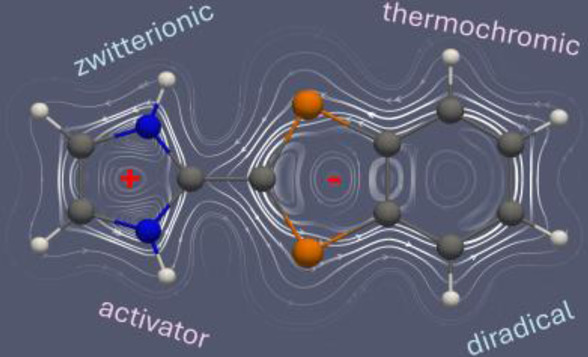

## Introduction

**Scheme 1:**
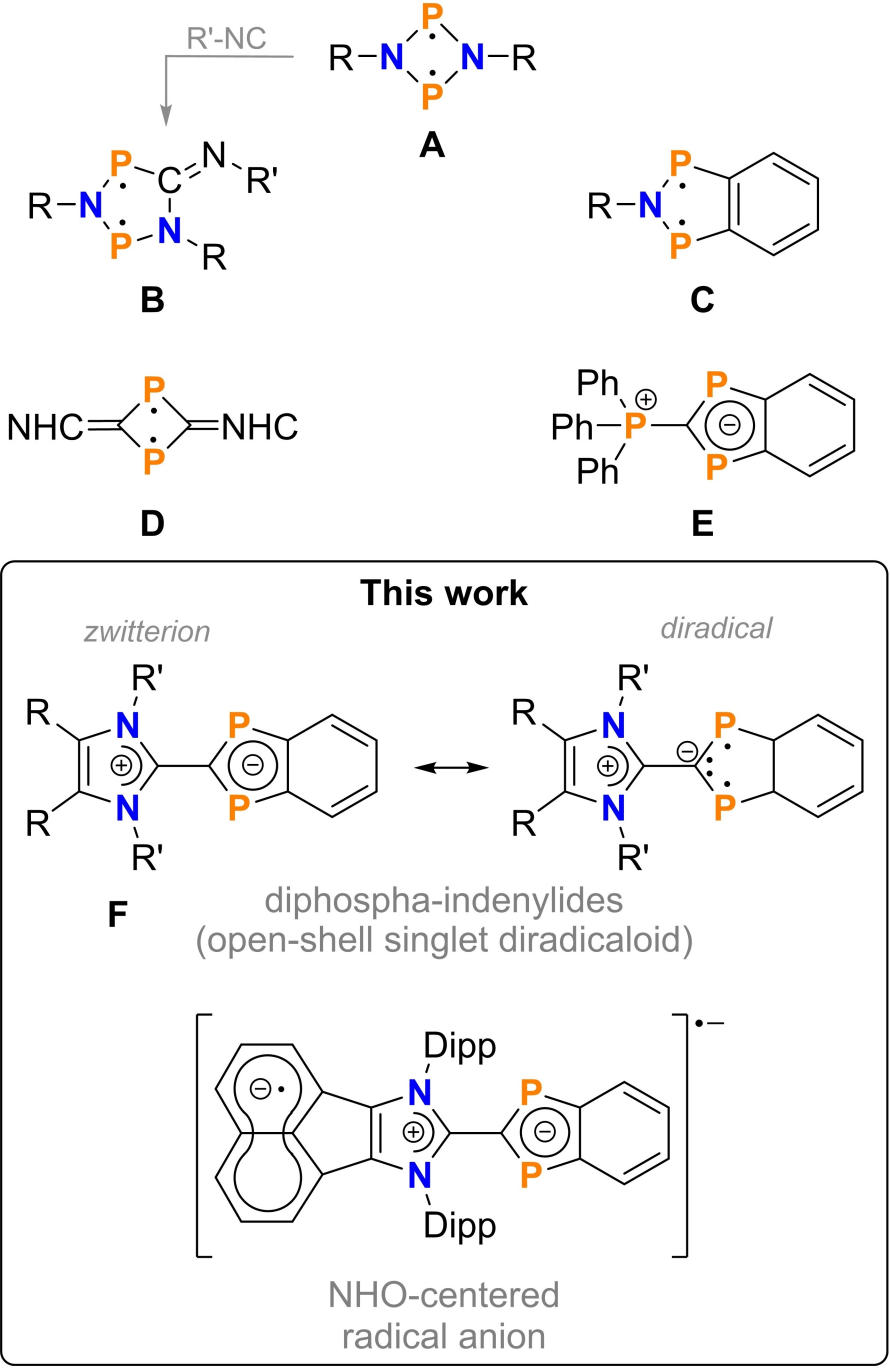
Examples of P‐centered diradicaloids related to this work. (NHC=*N*‐heterocyclic carbene, NHO=*N*‐heterocyclic olefin, Dipp=2,6‐^*i*^Pr_2_C_6_H_3_, R and R’ organic substituent)

Molecules with two electrons in two nearly degenerate orbitals are referred to as singlet diradical(oid)s.[^1^, ^2^, ^3^, ^4^, ^5^, ^6^, ^7^] These species have a net‐zero spin density at every point in space, however, their reactivity is generally enhanced compared to closed‐shell molecules.^[6]^ Stable main‐group centered diradicaloids have evaded their status as laboratory curiosities and have emerged as an attractive target for their potential in small‐molecules activation. Our group has carried out in‐depth studies on the phosphorus‐centered four‐membered ring diradicaloid [^**⋅**^P(*μ*‐NTer)]_2_ (Scheme 1 species **A**, R=Ter=2,6‐Mes_2_‐C_6_H_3_), which is highly reactive towards non‐polar and polar single and multiple bond systems (e.g. H_2_, CH_3_Cl, CO, CO_2_, alkenes, and alkynes) giving addition products with tri‐ or pentavalent phosphorus atoms.[^7^, ^8^]

**Scheme 2:**
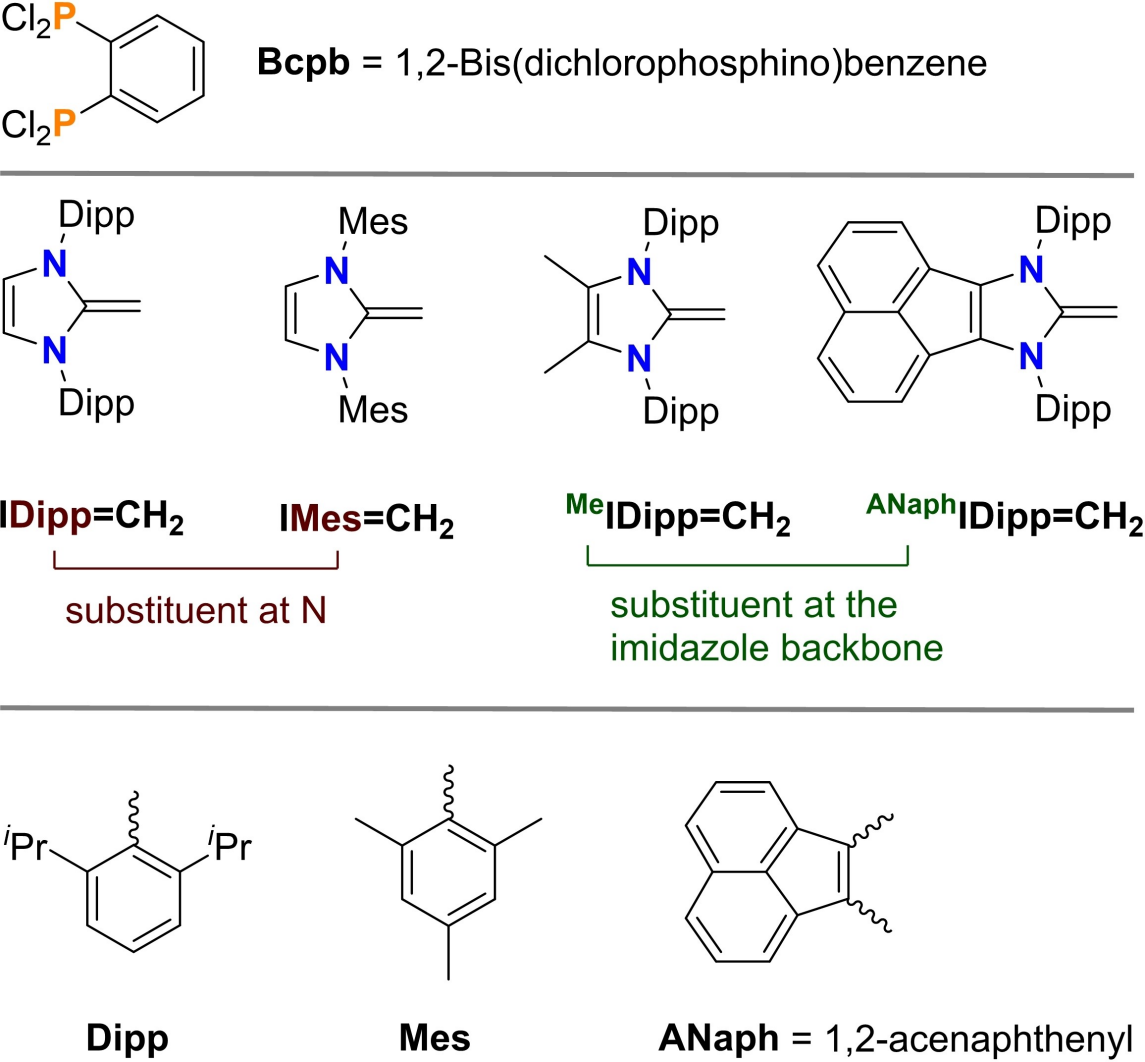
Bis(dichlorophosphino)benzene and substituents used to generate different NHOs.

For example, using isonitriles ring expansion reaction from hetero‐atom cyclobutane‐1,3‐diyls (**A**) to cyclopentane‐1,3‐diyls (**B**) have been observed.^[9]^ Light‐induced through‐space bond formation to the corresponding closed‐shell housane‐type isomer can be thermally reversed, making **B** candidates for the rational design of molecular switches.[^10^, ^11^, ^12^] Nevertheless, the follow‐up chemistry of **B** is hampered by reversibility of the initial CNR insertion step.[^6^, ^13^, ^14^] To increase the stability of diphospha‐cyclopentane‐1,3‐diyls (**B**) we have recently shown that benzofused azadiphosphaindane‐1,3‐diyls (**C**) are synthetically feasible using sterically demanding anilines in combination with 1,2‐bis(dichlorophosphino)benzene (**Bcpb**, Scheme 2).[^15^, ^16^] Oligomerization of the diradical intermediate was observed when smaller groups on the nitrogen were used.^[6]^

Ghadwal and co‐workers have recently shown that the base‐assisted dehydrohalogenation of NHO ^H^IDippCH_2_ (Scheme 2) with PCl_3_ yields a four‐membered phosphenium species that can be reduced to the P‐centered diradical [^**⋅**^P(*μ*‐C^H^IDipp)]_2_ (Scheme 1, species **D**, notation of ^H^IDipp see Scheme 2).^[17]^ From this, we reasoned that NHOs are suitable precursors for accessing open‐shell singlet diphospha‐indenylenides (**F**). NHOs formally contain an alkylidene unit terminally appended to a (mostly) formal *N*‐heterocyclic carbene (NHC) framework,[^18^, ^19^, ^20^] which is related to the methylidene‐phosphoranes, the classic Wittig‐reagents.[^21^, ^22^, ^23^] The olefinic C−C bond, particularly its π‐component, is strongly polarized towards the exocyclic C‐atom. In contrast to NHCs, NHOs are strong sigma‐donors with minimal to no π‐accepting properties. Due to its NHC‐backbone, NHOs can be readily modified in their steric and electronic properties, making them ideal candidates to easily tune diphospha‐indenylides derived from NHOs.

Here, we report the synthesis and full characterization of NHO‐based diphosphorus‐heterocycles which can be best referred to as open‐shell singlet diphospha‐indenylides (Scheme 1, species **F**). These NHO‐based diphospha‐indenylides with their unique structural and electronic characteristics represent a new class of heterocyclic compounds which, similar to the *P*‐ylide system described by Coburger et al.^[24]^ (Scheme 1, species **E**), exhibit zwitterionic character due to charge separation between the *N*‐ and *P*‐heterocyclic ring systems besides diradical character at the phosphorus atoms. Since the NHOs can be varied both in the imidazole backbone as well as on the two *N*‐linked substituents, the use of imidazole‐based NHOs with a terminal CH_2_ group enables customized design of the NHO‐linked diphospha‐indenylides. In this regard, it is worthy to note that the charge separation between the *N*‐ and *P*‐heterocyclic ring systems (with a positively charged imidazolium and a negatively diphospha‐indenylide) is complementary to the intrinsic zwitterionic character of singlet diradicaloids, i.e. the admixture of Lewis resonances of the type P⋅ ⋅P ↔ P^+^ P^−^ ↔ P^−^ P^+^ due to instantaneous electron‐electron correlation.[^2^, ^7^] We have investigated to what extent the zwitterionic and diradical character change when the substituents on the NHO are altered, and how they are affected by rotation around the central C−C axis (destabilized C−C π‐bond in the NHO) that connects the *N*‐heterocyclic ring to the diphospha‐indenyl heterocycle.

## Results and Discussion

Since our goal was to combine a diradicaloid with an imidazole‐based NHO, containing a terminal CH_2_ group, we first had to synthesize the corresponding NHOs with different bulky substituents (Scheme 2) and the 1,2‐bis(dichlorophosphino)benzene (**Bcpb**). *Note*: For better readability, we labelled all NHO species according to ^**R**^ **IR′** with I=imidazole, R=substituent in the backbone of the imidazole heterocycle, and R′=bulky substituent attached to both N atoms of the imidazole.

To generate stable NHO‐linked diradicaloids of type **F** (Scheme 1), several aspects must be considered in the design. Firstly, to prevent oligomerization, it needs sufficiently large steric protection, which can be tuned via the substituent at the nitrogen atoms and in the backbone of the imidazole heterocycle. Starting from *N*,*N*′‐substituted ethane‐1,2‐diimines, four differently substituted NHOs of the type ^**R**^ **IR′=CH_2_** (Scheme 2) were synthesised in multistep syntheses according to slightly modified synthetic routes known from the literature (see ESI).[^25^, ^26^, ^27^, ^28^, ^29^] NHO ^ANaph^IDipp=CH_2_, which appears blue due to π‐π* excitation within its π‐extended backbone (vide infra), was selected for its potential to facilitate electron delocalization upon product formation.

Secondly, bis‐dichlorophosphane **Bcpb** (Scheme 2, top), which had been previously used to access diradicaloids of type **C** (Scheme 1),^[15]^ was identified as a suitable precursor for the construction of the PCP diradicaloid subunit appended to a benzene ring (Scheme 1, species **F**). **Bcpb** is easily prepared either in a three‐step reaction sequence starting from 1,2‐dibromobenzene and *n‐*BuLi / ClP(NEt_2_)_2_[^15^, ^30^, ^31^, ^32^] or by a photo‐Arbuzov reaction with P(OMe)_3_ and subsequent reduction and chlorination.^[31]^

### Synthesis of NHO‐linked Dichlorodiphosphaindanes

**Scheme 3:**
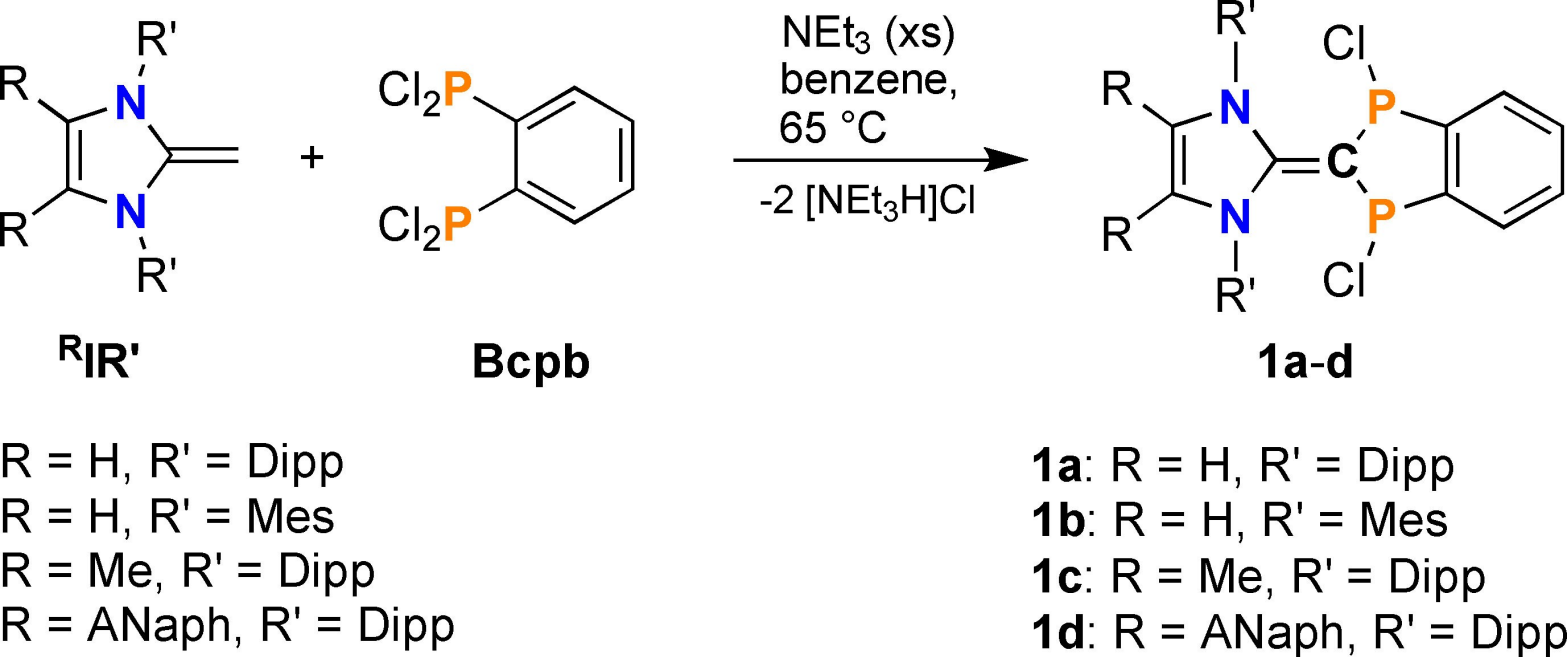
Synthesis of ^R^IR’=C(PCl)_2_C_6_H_4_ (**1**).

**Figure 1:**
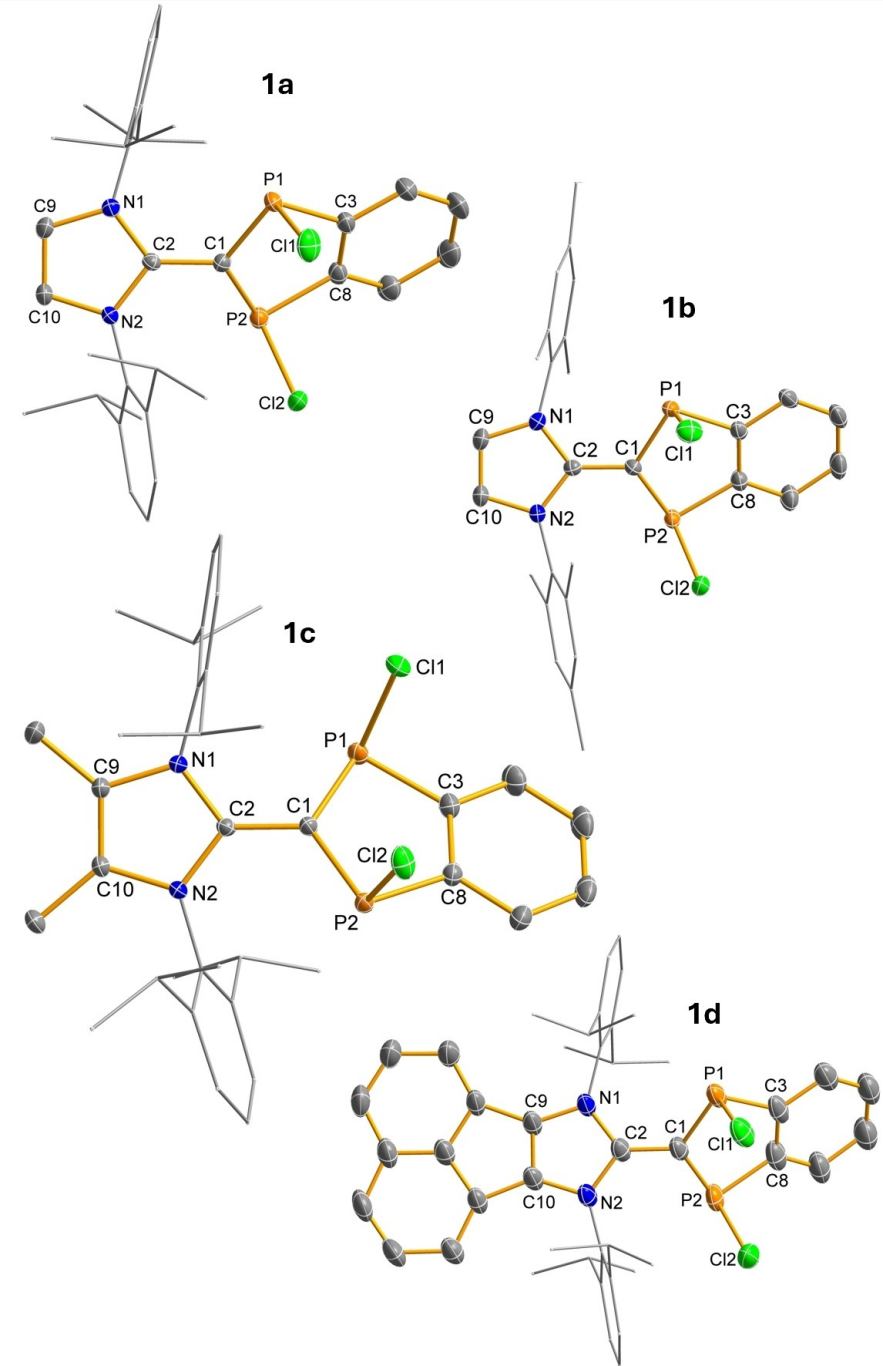
Molecular structures of **1a‐d** in the crystal (*T*=123(2) K, ellipsoids at 50 % probability). R’ substituent shown as wireframe. H atoms omitted for clarity. Selected bond lengths and angles are listed in Table 2.

With pure bis‐dichlorophosphane **Bcpb** and NHO (^**R**^ **IR′**) in hand, P−C ring formation reactions were carried out via elimination of two equivalents of HCl (Scheme 3). For this purpose, **Bcpb** dissolved in benzene was added to a solution of NHO (^**R**^ **IR′**) and NEt_3_ in benzene, and stirred for several hours at 65 °C. After filtration and recrystallization from benzene, the crosslinking products ^R^IR’=C(PCl)_2_C_6_H_4_ (**1a‐d**) could be isolated in good to very good yields as single‐crystalline materials (Figure 1 and Table 1). Compounds **1a‐d** are thermally stable up to well over 200 °C but should be stored under argon as they are sensitive to moisture. As expected, exactly one signal in the range 120 ‐ 125 ppm can be observed in the ^31^PNMR spectrum. Interestingly, a second, much weaker signal is found for **1b** at 164 ppm, which prompted us to investigate the structure of this compound in more detail. In accordance with calculated ^31^P NMR and structural data, this is a mixture of *cis*‐*trans* isomers, with the *cis* isomer being the thermodynamically favoured isomer (Δ*G*°(*trans‐cis*)=4.4 kJ/mol for **1b**; cf. 7.8 kJ/mol for **1a**). In the case of **1a**, **1c** and **1d**, only the *cis* isomers are observed in solution as well as in the solid state (Scheme 3). Single crystal X‐ray diffraction (SCXRD) studies revealed exclusively the presence of *cis* isomers, and a planar imidazole ring bound to a slightly folded P_2_C_3_ heterocycle for all species considered [Figure 1, ∢(C3−P1−C1−P2) between 5° (**1a**) and 15° (**1d**)]. The two five‐membered heterocycles are rotated around the C1−C2 axis by approx. 23 ‐ 31° relative to each other. While the P−C distances of 1.76 ‐ 1.77 Å are all in the range of a typical polarized P−C single bond (cf. Σ*r*_cov._(C−P) = 1.86 Å, 1.764 Å in **E**),^[24]^ the C1−C2 bond lengths (1.41 ‐ 1.43 Å)^[33]^ are in the range between a C−C single and double bond (cf. Σ*r*_cov._(C−C) = 1.50, Σ*r*_cov._(C=C) = 1.34 Å).^[33]^

**Table 1:** Selected experimental data of ^R^IR’=C(PCl)_2_C_6_H_4_ (**1**) and ^R^IR’=CP_2_C_6_H_4_ (**2**)

compound	Color	Yield [%]	*T*_M_ [°C]^[c]^	δ[^31^P]
**1a**	yellow^[g]^	72	253^[e]^	121.7^[a]^
**1b**	yellow	48	283^[e]^	121.4^[a]^ 164.2^[b]^
**1c**	yellow	85	278^[e]^	125.5^[a]^
**1d**	red	68	308^[e]^	120.0^[a]^
**2a**	yellow^[c]^	82	287^[d]^	195.9
**2b**	orange^[c]^	57	355^[d]^	189.6
**2c**	yellow^[c]^	76	364^[d]^	196.6
**2d**	red	18	374^[d]^	200.8
**3d**	red	46	158^[e]^	^[f]^

[a] *cis*‐isomer (see Scheme 3). [b] *trans*‐isomer, ratio *cis* : *trans*=25 : 1. [c] at 298 K, substance is thermochromic. [d] average value. [e] decomposition, [f] paramagnetic, [g] in the bulk phase yellow, under the microscope, the single crystals appeared yellow‐green (Table S2).

### Synthesis of NHO‐linked Diradicaloids

**Scheme 4:**
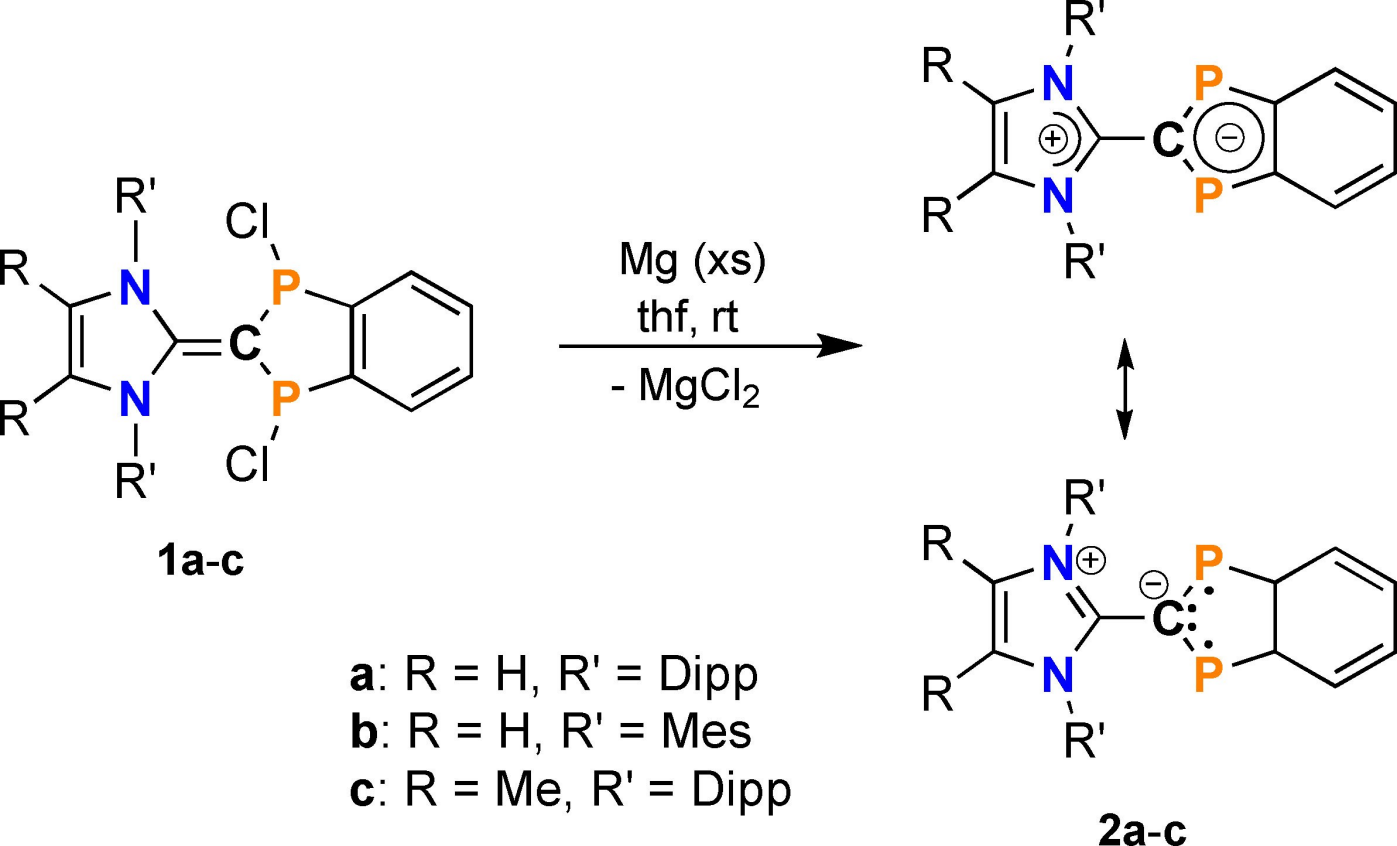
Synthesis of ^R^IR’=C(P)_2_C_6_H_4_ (**2**).

After we had successfully linked the NHOs to the bisphosphane via the terminal C atom, we investigated the reduction of the P−Cl bonds in **1a‐d** in a second series of experiments utilizing different reducing agents (Scheme 4). Both KC_8_ and Mg chips were used as reducing agents, whereby it was shown that the reductions with Mg proceeded more slowly, but fewer by‐products were produced, so that better yields could be achieved (see ESI for details). The reductions were carried out at room temperature by adding mechanically activated Mg chips to a solution of **1a‐c** in THF and stirring for about 1 ‐ 2 h. During the reduction, the solution turned red within a few minutes and the reaction could be monitored by ^31^P NMR spectroscopy since a significant downfield shift of the product signals was observed (cf. δ[^31^P], **1a‐c**: 121 ‐ 126 vs. **2a‐c**: 189 ‐ 196 ppm). After recrystallization from benzene at 80 °C, **2a‐c** were afforded as orange solids in good yields (60 ‐ 80 %, Table 1). Compounds **2a‐c** are very sensitive to moisture and oxygen but can be stored sealed under argon in an ampoule over a long period of time (Scheme 4). Interestingly, all three compounds show thermochromism, i.e. they are yellow or orange at low temperatures, while at higher temperatures a color change to deep red occurs, which is observed near the melting point and in the melt (vide infra). Moreover, all three yellow/orange compounds dissolve with red color in THF, benzene or CH_2_Cl_2_ (Figures S48 and S49).

**Scheme 5:**
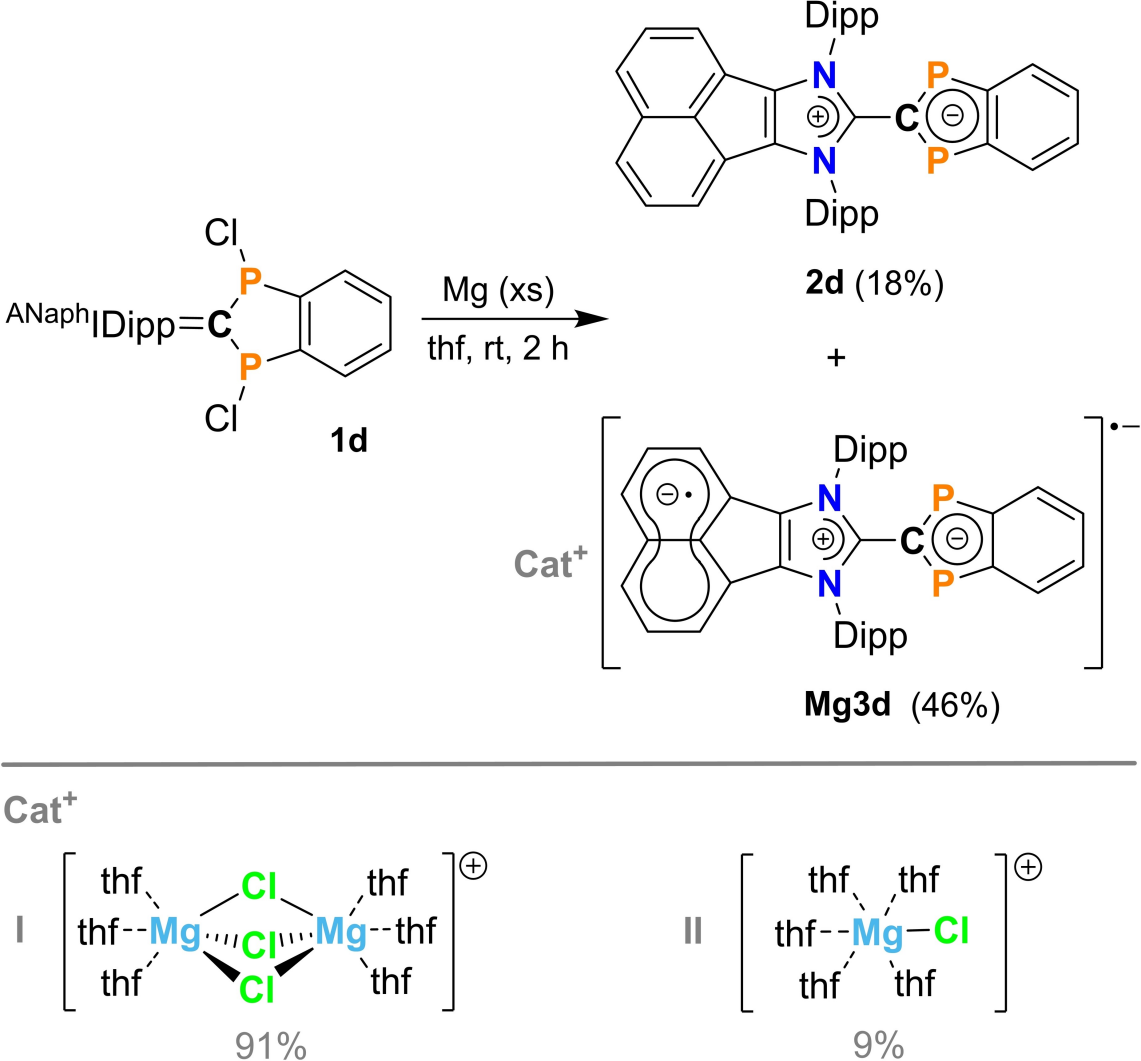
Synthesis of ^ANaph^IDipp=C(P)_2_C_6_H_4_ (**2d**) and Mg‐salt of **3d^⋅–^** (simplified notation: **Mg3d**).

In contrast to the reduction of **1a‐c** with Mg, the reduction of **1d** is not as simple, as over‐reduction cannot be suppressed. This means that a product mixture of the desired product **2d** and a magnesium salt with the unusual radical anion **3d^⋅–^** is always formed (Scheme 5). However, both species can be separated by fractional crystallization from benzene as red crystalline solids, which show no thermochromism.^[34]^ While crystals of **2d**, dissolved in benzene, show a singlet resonance in the ^31^P NMR spectrum at 200.8 ppm, compound **Mg3d** bearing the radical anion **3d^⋅–^** is NMR‐silent. Therefore, EPR investigations were carried out to prove the presence of a radical anion (vide infra). Both compounds, **2d** and **Mg3d** are not thermochromic (see detailed discussion of UV spectroscopic experiments and computations below).

### Structure and Bonding of Diradicaloids 2

**Figure 2:**
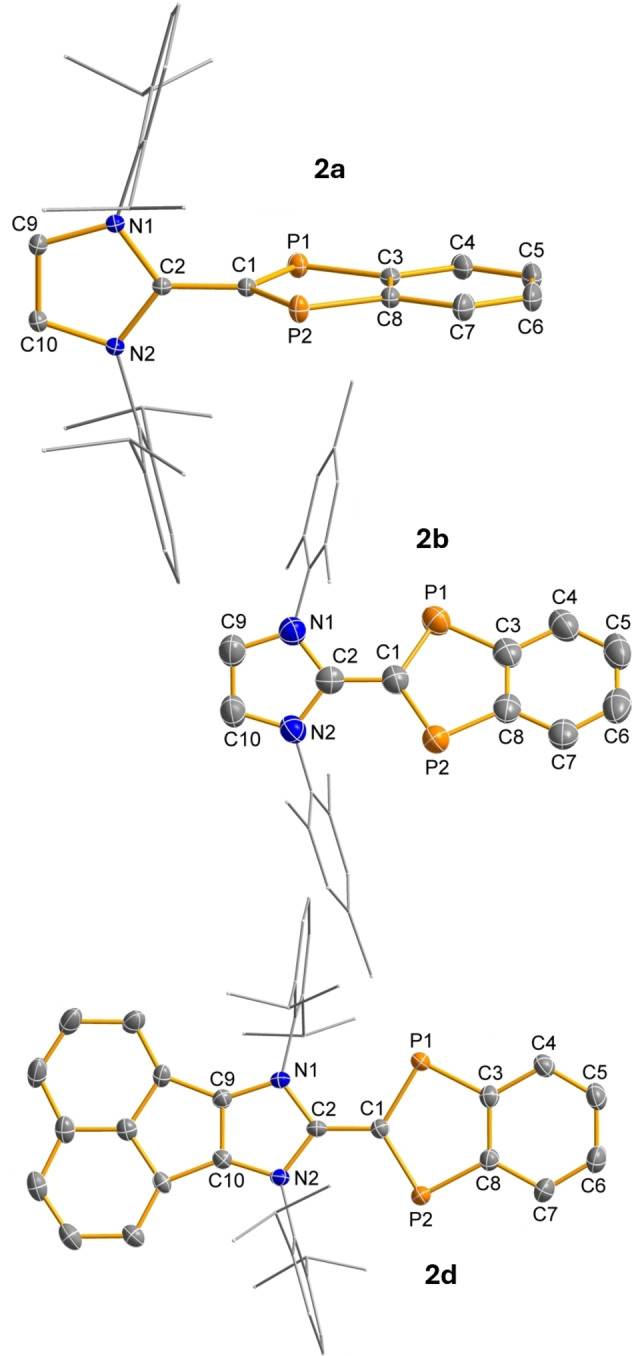
Molecular structures of **2a**, **2b**, and **2d** in the crystal (*T*=123(2) K, ellipsoids at 50 % probability). R’ substituent shown as wireframe. H atoms omitted for clarity. Selected bond lengths and angles are listed in Table 2.

*SCXRD study*. Single crystalline material suitable for SCXRD experiments was obtained by recrystallization from benzene (**2a**, **2b**, and **2d**) or THF (**2d**). For compound **2c**, only microcrystalline material could be obtained, which was not suitable for SCXRD studies. The molecular structures are shown in Figure 2. Crystallographic details are listed in Tables S2 ‐ S7 and selected structural parameters in Table 2. It should be noted that **2a**, which was recrystallised from benzene, crystallized solvent‐free, whereas **2b** and **2d** crystallized with benzene molecules (**2d⋅2C_6_H_6_**, not shown in Figure 2). Solvent‐free single crystals of **2d** were obtained by recrystallization from THF. In all structures there are no significant intermolecular interactions, since all species are sterically protected by a sufficiently large substituent R′ on both nitrogen atoms, which prevents dimerization of these diradicaloids, as is frequently observed.[^8^, ^15^] As shown in Figure 2, the molecular structure of all species **2** consists of two planar rings, the P‐substituted indenyl heterocycle and the substituted imidazolyl ring. Both rings are connected to each other via a C−C bond (C1−C2). All C1−C2 bonds are slightly elongated compared to the dichlorodiphosphaindane precursors **1**, but still exhibit partial double bond character (1.43 ‐ 1.46 Å; Σ*r*_cov._(C−C) = 1.50  and Σ*r*_cov._(C=C) = 1.34 Å;^[33]^ cf. 1.380(3) Å in **D**).^[17]^ All other structural parameters do not change significantly compared to **1**, as shown in Table 2, with one exception, the dihedral angle between the two linked five‐membered heterocycles [*D*=∢(P1−C1−C2−N1)]. Using different substitution patterns, it was possible to set the angle between the two heterocycles to almost 0° (**2d**: *D*=5°) and 90° (**2a**: 88°) or between these two limits (**2b**: 33°). Interestingly, the P1−C1−C2−N1 dihedral angle in **2d⋅2C_6_H_6_** is clearly different at 24° from 5° in **2d**, which prompted us to take a closer look at this phenomenon (vide infra).

**Table 2:** Selected experimental bond lengths (Å) and angles (°) of **1a‐d**, **2a‐d**, **3d^⋅–^**, and **Xa** (X=**4**‐**7**).

parameter	**1a**	**1b**	**1c**	**1d**
P1−C1	1.769(2)	1.773(1)	1.7662(8)	1.764(2)
P1−P2	3.027(1)	3.0273(5)	3.0124(5)	3.017(4)
C1−C2	1.424(3)	1.428(2)	1.428(1)	1.416(3)
P1−C1−P2	118.0(1)	117.82(7)	117.13(4)	118.3(2)
P1−C1−C2	122.7(2)	120.3(1)	118.90(6)	121.2(2)
*D* ^*[a]*^	29.7(4)	23.6(2)	34.5(1)	30.7(3)
**parameter**	**2a**	**2b**	**2d**	**3d^⋅–^**
P1−C1	1.746(1)	1.755(3)	1.769(1)	1.759(3)
P1−P2	3.0173(5)	3.019(1)	3.0229(7)	3.003(3)
C1−C2	1.455(2)	1.443(4)	1.434(2)	1.461(3)
P1−C1−P2	119.42(7)	118.7(2)	117.15(8)	117.7(2)
P1−C1−C2	117.23(9)	120.8(2)	121.0(1)	122.7(3)
*D* ^[a]^	–87.8(1)	–33.0(5)	5.2(2) ^[b]^	–59.9(6)
**parameter**	**4a**	**5a**	**6a**	**7a**
P1−C1	1.809(1)	1.810(3)	1.808(4)	1.821(1)
P1−P2	2.8578(5)	2.870(1)	2.866(2)	2.8728(6)
C1−C2	1.382(2)	1.363(4)	1.392(4)	1.376(2)
C10−C11	1.347(2)	1.331(5)	1.542(5)	1.557(2)
P1−C1−P2	104.48(7)	104.5(2)	104.8(2)	104.46(7)
P1−C1−C2	127.3(1)	127.6(2)	126.8(3)	126.9(1)
*D* ^[a]^	13.6(2)	17.6(4)	‐16.5(5)	–1.1(2)

[a] Dihedral angle: ∢(P1−C1−C2−N1). [b] –24.0(5)° in **2d⋅2C_6_H_6_**, all other parameters are very similar.

*Electronic structure ‐ diradical* versus *zwitterion*. MO‐ and VB‐type methods were used to shed some light on the electronic structure of the differently substituted diradicaloids with a specific focus on the question of which is more dominant, the diradical or the zwitterionic character. We started with model compound **2H** in which all substituents R and R’=H. Since it was assumed that the diradical character changes with the P−C−C−N dihedral angle, as the formal C−C double bond is broken at 90° and both five‐membered heterocycles can no longer interact significantly with each other in the region of the π bond, NBOs[^35^, ^36^, ^37^] and NRT[^38^, ^39^] Lewis representations were calculated for three different angles, namely 0°, 36.1° (optimized dihedral angle of **2a**) and 90° (Figures S87 ‐ S93, NBO=natural bond analysis, NRT=natural resonance theory).

**Scheme 6:**
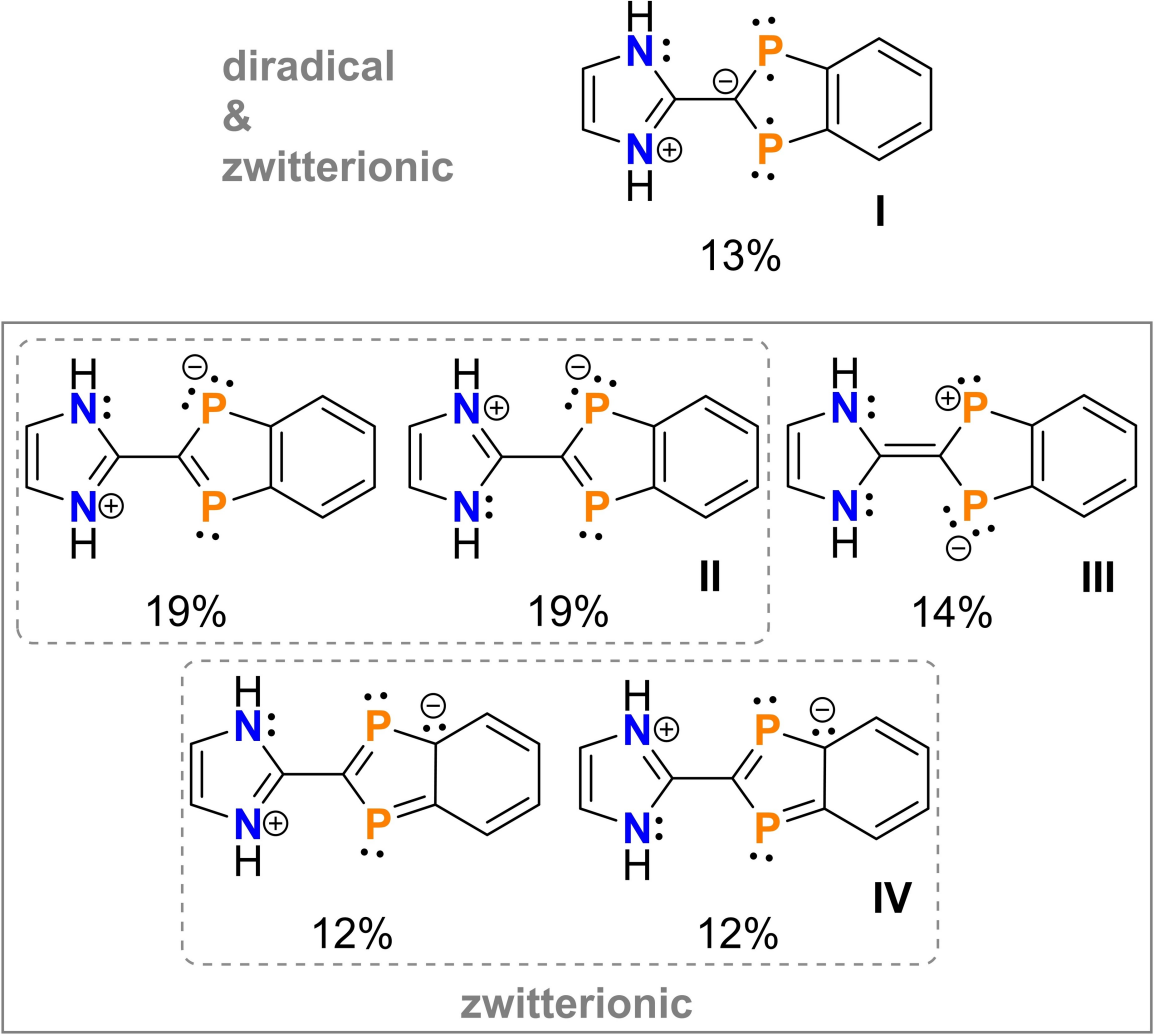
Leading resonance structures of model compound **2H** (*D*=0°) according to NRT calculations. [*D*=Dihedral angle: ∢(P1−C1−C2−N1); PBE0‐D3/def2‐TZVP, shown only for those resonances with a weight >4 %. Only one structure of symmetry‐equivalent resonances is depicted, the sum of the individual weights is reported.]

From these calculations it can be concluded that (i) the diradical character decreases from 0 to 90° in agreement with CASSCF calculations (vide infra) and (ii) zwitterionic Lewis formulae without unpaired electrons (**II‐IV**) have a greater weight than the diradical‐type structure (**I**) with two unpaired electrons, as shown in Scheme 6 for a dihedral angle *D*=0°. Moreover, the model calculations nicely demonstrate that diradical and zwitterionic character actually coexist in a single Lewis representation **I** due to the charge separation between the *N*‐ and *P*‐heterocycles, as opposed to the zwitterionic character between the two P atoms (**III**) which does not result in an overall charge separation (note that there are two symmetry‐equivalent resonances of type **III**, i.e. the formal charges may also be reversed).

Both NBO and NRT analyses find Lewis representation **II** as the energetically most favorable Lewis structure for **2H**. Structure **II** describes a zwitterion with the positive formal charge localized at the N atoms (two resonance formulae each), while the negative formal charge is localized at the phosphorus atoms in the indenyl heterocycle. This picture is supported by the calculation of the charge transfer between both rings, which amounts to 0.64e. That is, the molecule might be regarded as a zwitterion composed of an imidazolium and a diphospha‐indenylide fragment. The charge transfer occurs from the imidazolyl into the indenyl ring and increases from 0° to 90° (0.74e, Table S22). Thus, at 90°, the charge separation is maximized, due to the absence of π‐conjugation between the two oppositely charged heterocycles. In accordance with the relatively long C1−C2 bond length, the Lewis formulae with a C1−C2 double bond have only a relatively low weight.

Zwitterionic structure **I** for **2H** formally describes a P‐centered diradical with a benzene linker that has delocalized 6π electrons. Together with one lone pair of electrons on the C atom, and the two unpaired electrons at the P atoms, all of which are localized in p‐atomic orbitals, this results in a total of 10π electrons for the indenyl heterocycle. Formal charges arise because there is no C1−C2 double bond, but rather a lone pair of electrons on the C(indenyl) atom (C1) and an N−C(imidazolyl) double bond, which ultimately leads to the formation of a zwitterion. Therefore, the best description in the VB picture is certainly the resonance between structure **I**, **II** and **IV**, i.e. without a C−C double bond between the two heterocycles, and compound class **2** can best be described as a zwitterion with a diradical component.

**Figure 3:**
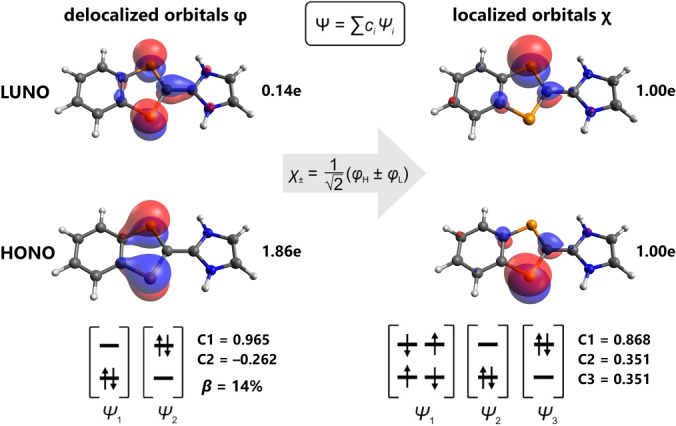
CASSCF(2,2)/def2‐TZVP orbitals of **2H** (*D* = 0°). The orbital occupation numbers of the CASSCF calculation are given (isovalue=0.05). CASSCF orbitals of **2a**‐**2d** are shown in Figures S62 ‐ S65.

Different series of CASSCF(2,2)/def2‐TZVP computations were carried out to study the open‐shell character (see ESI). First the structures of all four species **2a**‐**d** were calculated at the PBE‐D3/def2‐TZVP level of theory. Interestingly, for all species P−C−C−N dihedral angles between 36.6° (**2b**) and 30.6° (**2c**) were found. Apparently, the deviations in the solid with respect to the calculated dihedral angles are due to dispersion interactions between the molecules and a flat potential energy surface for rotation around the central C−C bond. However, since the diradical character of **2** depends on the P−C−C−N dihedral angle, this dependence (in addition to that on substitution) also needed to be investigated. Secondly, CASSCF(2,2)/def2‐TZVP computations (Figure 3) indicated for all four species (including **2H**) an open‐shell singlet ground state with a diradical character in the range 12 ‐ 14 % ($\beta =2c_{2}^{2}/\left(c_{1}^{2}+c_{2}^{2}\right),$ *c*_i_=coefficients of CASSCF wavefunction, Figures S62 ‐ S68; CASSCF = complete active space self‐consistent field, LUNO = lowest unoccupied natural orbital, HONO=highest occupied natural orbital).^[40]^ The HONO and LUNO are mainly localized at the indenyl heterocycle, the HONO being transannular antibonding and the LUNO transannular bonding between both P atoms, which are the typical characteristics of related phosphorus‐centered diradicaloids as depicted in Figure 3.[^6^, ^8^, ^16^] From these calculations we could conclude that the diradical character does not depend significantly on the substitution pattern, which is why we next examined the dependence on the dihedral angle in more detail.

**Figure 4:**
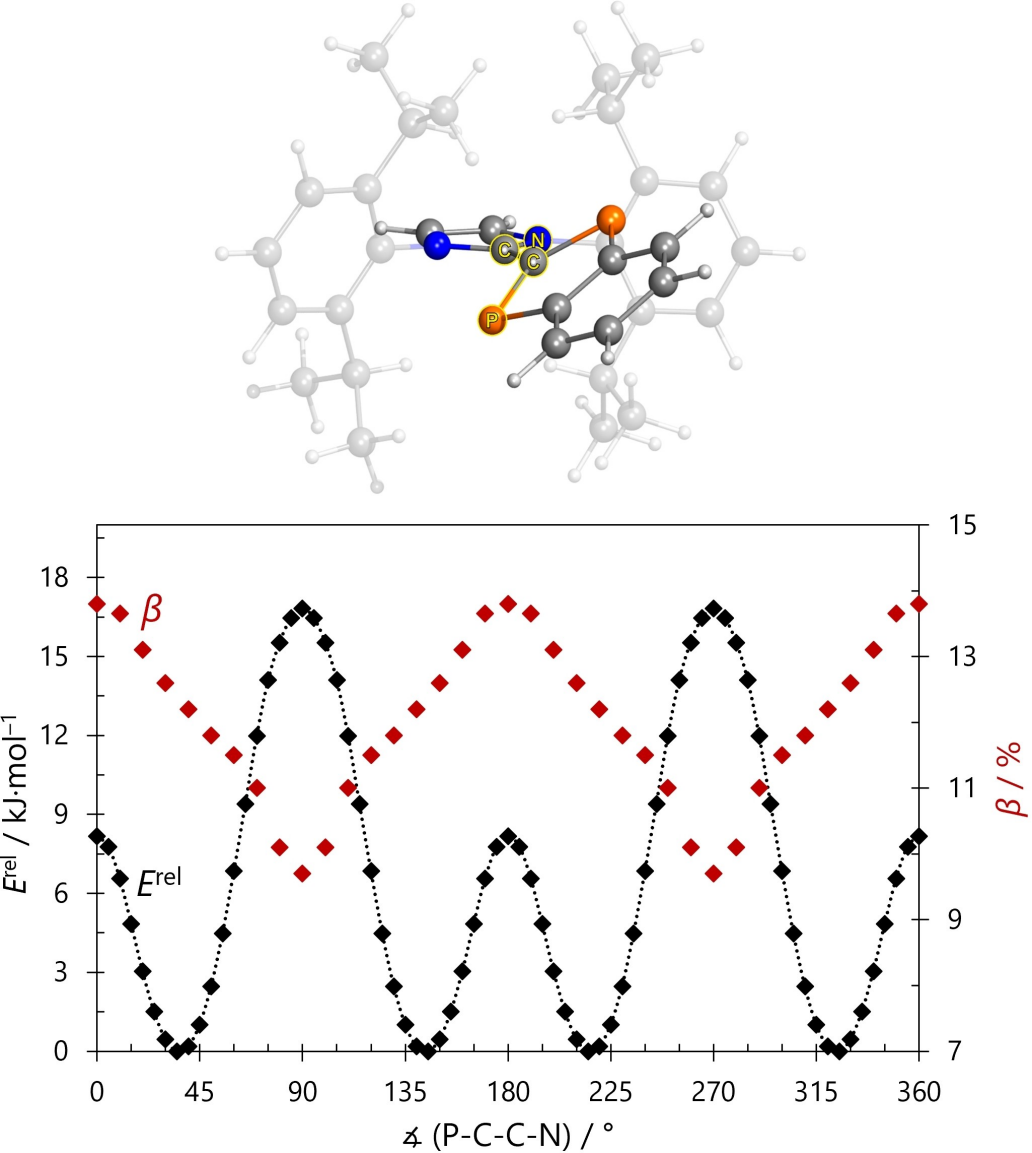
Top: Illustration of the P−C−C−N dihedral angle. Bottom: Relative energy as a function of the P−C−C−N dihedral angle (black) for **2a** and diradical character *β* as a function of the P−C−C−N dihedral angle (red).

To investigate the rotational barrier of **2a** and the dependence of the diradical character on the P−C−C−N dihedral angle (Figure 4), a relaxed potential energy surface scan (PBE‐D3/def2‐TZVP) was conducted with the dihedral angle as the scanned variable (step size 5°). For every second structure obtained, the diradical character was determined using CASSCF(2,2)/def2‐TZVP single point calculations. Two things can be deduced from this study (Figure 4): (i) The potential is very flat with rotation barriers smaller than 18 kJ/mol and there is only one true rotamer, as the two isomers at 36 and 144° are mirror symmetric. (ii) The diradical character decreases successively from 14 % (0°) to 9 % (90°), i.e., the diradical character decreases with increasing dihedral angle and charge separation (vide supra, Table S19). This clearly shows that the formal C−C π‐bond is highly polarized and cleaved heterolytically upon rotation, in agreement with the notion of an inner salt. This applies to all diradicaloid systems **2** considered here. As the rotational barrier is relatively small, the rotation in solution is not restricted at ambient temperatures, which is important for the interpretation of UV/Vis spectra and thermochromism (vide infra). In line with the decreasing diradical character with increasing dihedral angle (up to 90°), there is also a widening of the C−C distance (Figure S71) as expected for breaking of the C−C π‐bond.

*UV/Vis‐Spectroscopy – thermochromic behaviour*. To investigate the influence of the P−C−C−N dihedral angle on the color (thermochromism) of **2**, the UV/Vis spectra were calculated for dihedral angles ∡(P−C−C−N)=0°, 36.1°, and 90°, respectively, where 36.1° corresponds to the optimized structure at the PBE‐D3/def2‐TZVP level of theory (**2a**, Tables S23 ‐ S25 and Figures S74 ‐ S81).

**Figure 5:**
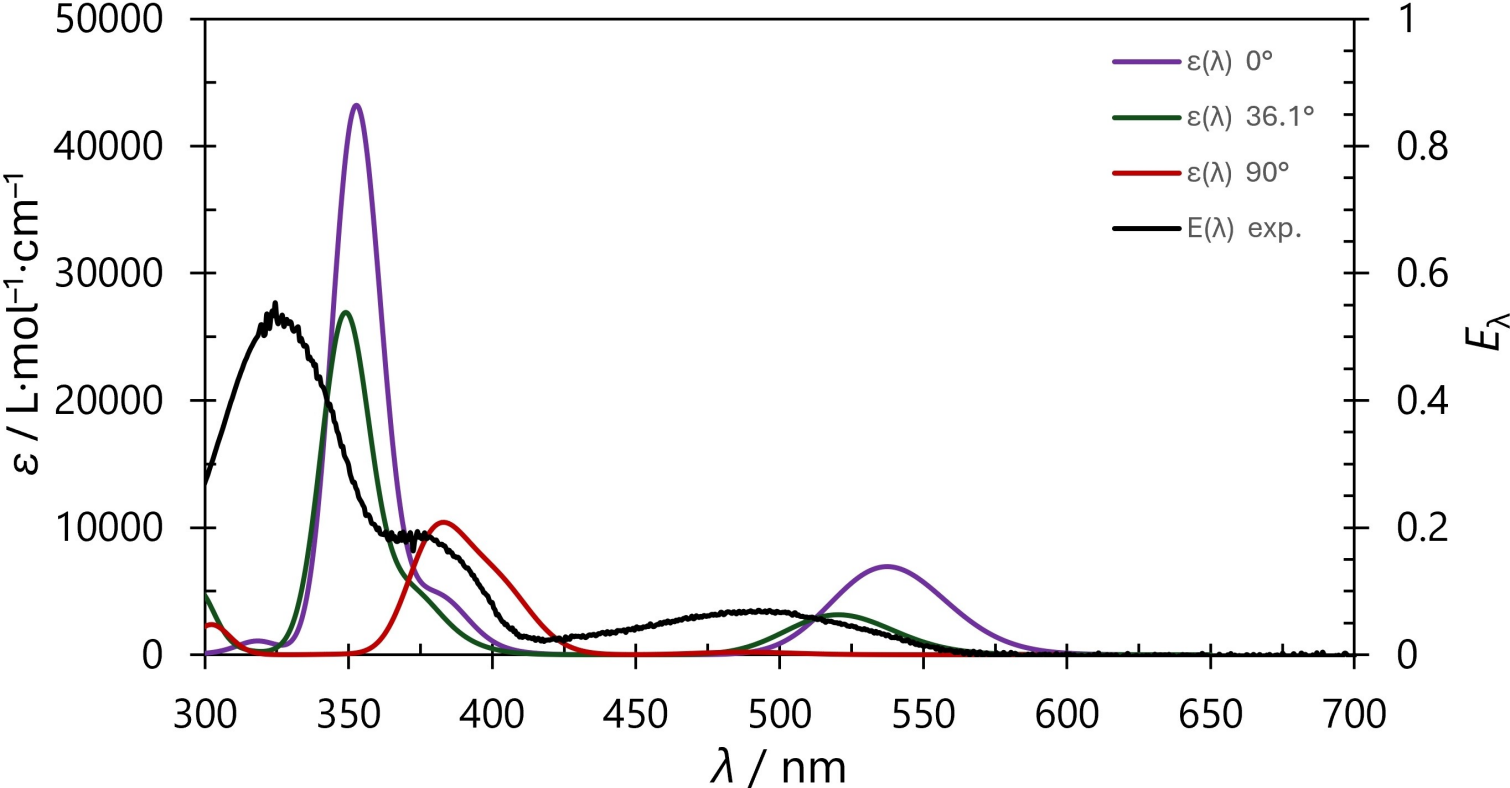
Comparison of the experimental and calculated UV/Vis spectra of **2a** (PBE0‐D3/def2‐TZVP).

**Figure 6:**
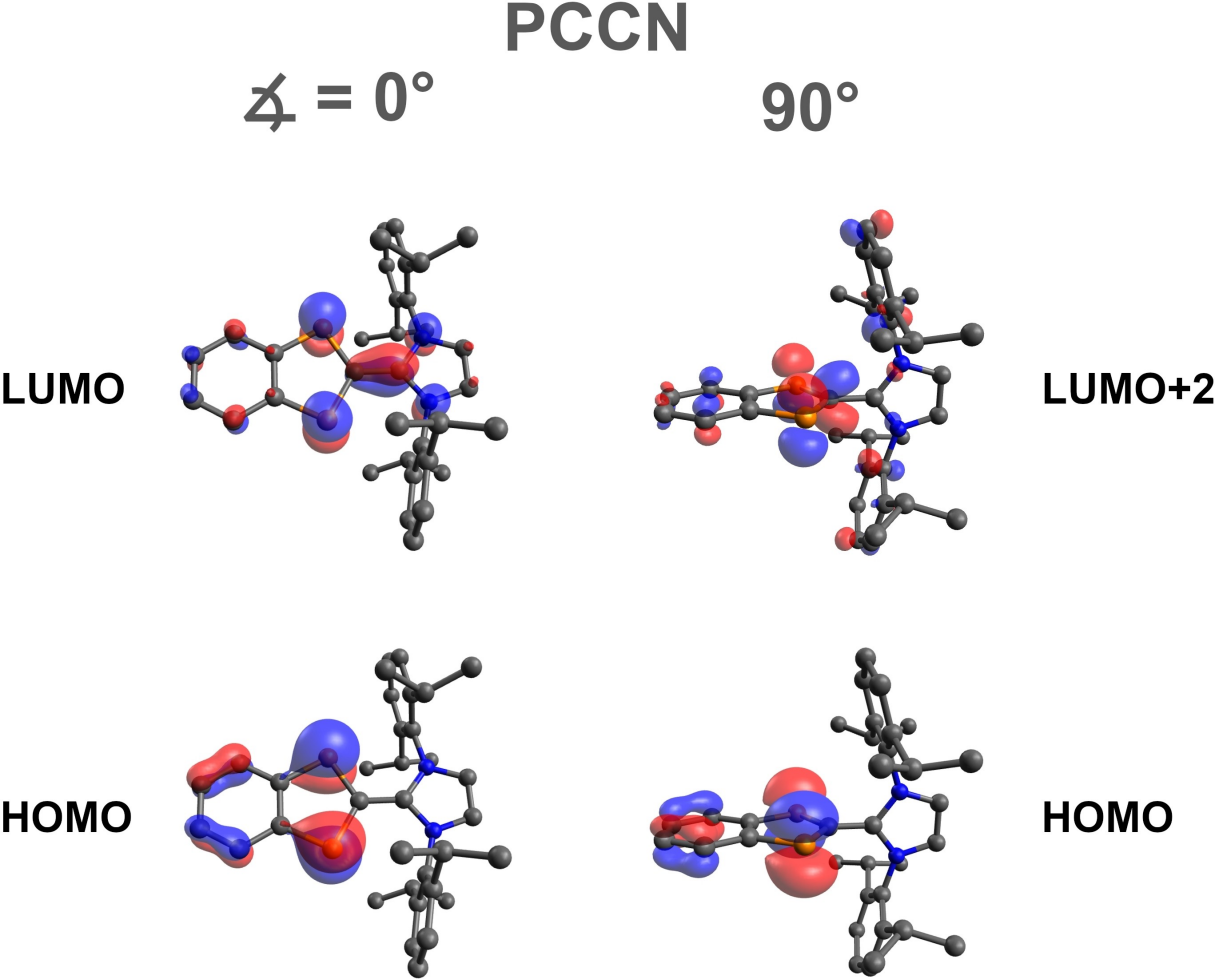
Frontier molecular orbitals of **2a** at different dihedral angles (PBE0‐D3/def2‐TZVP, isovalue=0.04), H atoms omitted for clarity.

Compound **2a** was taken as an example because it showed pronounced thermochromism (vide supra, Figure 5). TD‐DFT computations at the PBE0‐D3/def2‐TZVP level of theory revealed that the HOMO→LUMO transition is mainly responsible for the red color in solution (Figure 6). Experimentally, this transition is observed in the solution spectrum at room temperature at approx. 490 nm as a very broad band (black plot in Figure 5), in good agreement with calculated data (36.1°: 520 nm). Notably, the position of this band varies with P−C−C−N dihedral angle: At 0° the HOMO→LUMO transition was calculated at 537 nm, whereas at 90°, the analogous transition (now dominated by the HOMO→LUMO+2 transition due to a change in the order of the MOs, Figure 6 right) is predicted at 401 nm. Thus, the absorption shifts to smaller wavelengths with increasing dihedral angle (0°: 537, 36.1°: 520, 90°: 401 nm). Moreover, the calculated oscillator strength of the band decreases, so other bands at around 400 nm become relevant at a dihedral angle of 90° (cf. Figures S77 ‐ S80, Table S25). This nicely explains the yellow color in the solid state, since the molecular structure is fixed at a dihedral angle of 88° due to packing effects. As rotation is possible at higher temperatures (e.g. in the melt) or in solution, the absorption at approx. 520 nm dominates in this case and red color is perceived, explaining the thermo‐ and solvatochromism.

The change in energy of the LUMO depending on the dihedral angle can be traced back to varying delocalization of the orbital into the NHC moiety. In the case of ∡(P−C−C−N)=0°, the LUMO, which has bonding character between the two P atoms, is delocalized across the central C−C double bond into the imidazole ring system. This is no longer possible at 90° and the orbital is mainly localized on the diphospha‐indenylide ring system. Therefore, it is energetically raised (now LUMO+2) due to lack of delocalization. The HOMO remains virtually unchanged and corresponds to the P−P antibonding orbital (vide supra).

Finally, we would like to address why compound **2d** is *not* thermochromic. In contrast to thermochromic derivatives **2a‐c**, the LUMO of **2d** is not localized at the *P*‐heterocycle but rather at the 1,2‐acenaphthyl substituent in the backbone and is therefore not prone to change upon rotation around the central C−C axis (see NHO backbone in Figure S82). The same holds true for the radical anion **3d^⋅–^** (vide infra).

**Figure 7:**
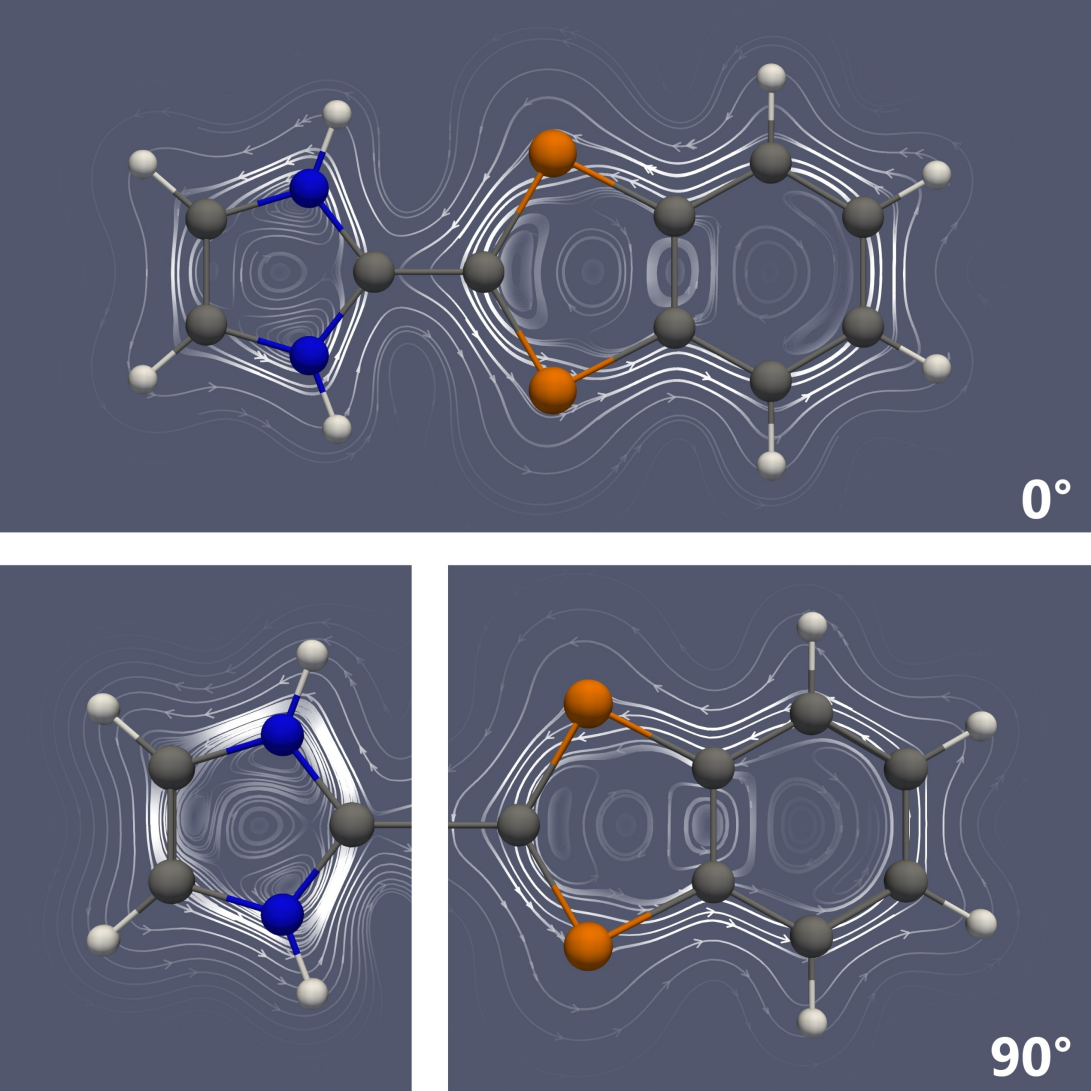
Streamline representation of the induced current density susceptibility of **2H** at P−C−C−N dihedral angles of 0° (top) and 90° (bottom). The streamlines are plotted in a plane 1 Bohr above the molecular plane. Since the two rings in the bottom structure are perpendicular to each other, the induced ring currents were calculated independently by subsequently aligning the magnetic field vector perpendicular to each of the ring systems.

*Aromaticity*. The variation of the diradical character depending on the P−C−C−N dihedral angle (vide supra) also prompted us to take a closer look at the aromaticity of the imidazolium‐diphospha‐indenylides. In particular, we decided to investigate the nucleus independent chemical shifts (NICS values)[^41^, ^42^] as well as magnetically induced current density susceptibilities[^43^, ^44^, ^45^, ^46^, ^47^] as two possible descriptors of aromaticity using the model system **2H** (R = R’ = H). Qualitatively, irrespective of the dihedral angle, we find a diatropic ring current within both the imidazolium as well as the diphospha‐indenylide moieties (Figure 7), in accordance with 6π and 10π electron systems, respectively, that are not significantly disturbed by π‐bonding between the two ring systems. As such, the aromaticity underpins the description of **2** as a zwitterionic species, containing a cationic imidazolium and an anionic diphospha‐indenylide subunit. On closer inspection, however, we find that the net induced current susceptibilities imply that the aromaticity is slightly less pronounced at 0° (imidazolium: 5.8, indenylide: 10.0 nA/T; cf. Table S31) and highest at 90° (imidazolium: 8.4, indenylide: 12.0 nA/T). Similar conclusions may be drawn, for example, from the NICS(1)_zz_ value, which increases from −21.1 (0°) to −28.0 ppm (90°) for the five‐membered P_2_C_3_ scaffold (cf. Table S31). These findings complement the results concerning the diradical character, which is lowest when the aromaticity is highest and vice versa. This is the expected behaviour:^[48]^ at 0° there is a small amount of π‐conjugation between the two sub‐units and therefore the aromaticity (i.e. delocalization *within* the ring systems) is somewhat disturbed, which in turn may promote diradical character (i.e. localization of the electrons on the P atoms) if some prerequisites are met such as a relatively small HOMO–LUMO gap. At 90°, on the other hand, there is no π‐bonding interaction between the two ring systems, and they are therefore maximally aromatic (i.e. the electrons are maximally delocalized) and the diradical character is therefore suppressed. Thus, the intricate interplay between π‐conjugation, aromaticity and diradical character may be exceptionally well studied in **2** due to the dependence of these parameters on the P−C−C−N dihedral angle.

### Radical Anion 3 d^–^

**Figure 8:**
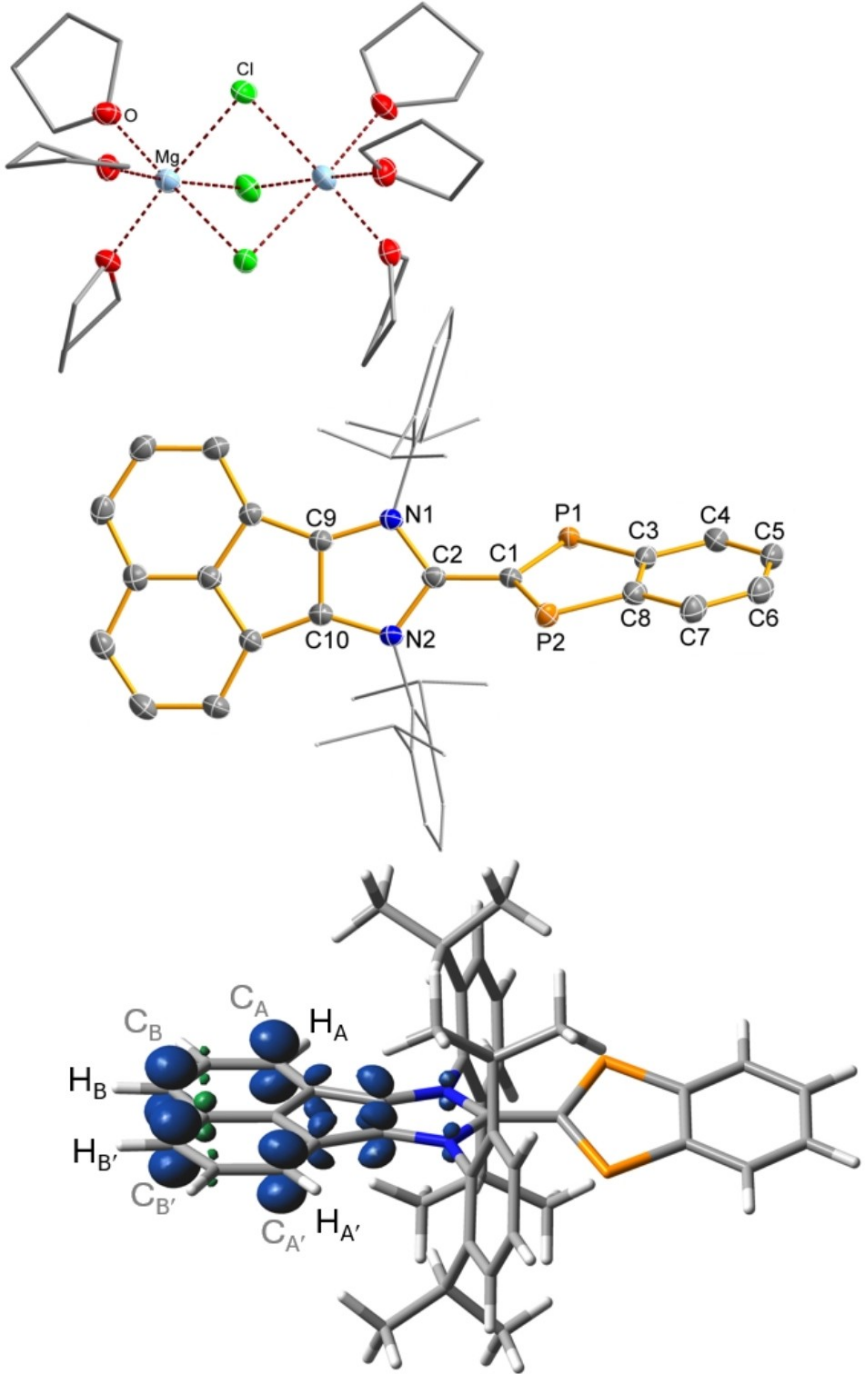
Top: Molecular structure of **Mg3d** in the crystal (*T*=123(2) K, ellipsoids at 50 % probability). H atoms omitted for clarity. Only cation **I** from Scheme 5, [Mg_2_Cl_3_(C_4_H_8_O)_6_]^+^, is shown. Selected bond lengths and angles are listed in Table 2. Bottom: Calculated spin density distribution of **3d^⋅–^** (PBE0‐D3/def2‐TZVP(D) single point calculation, isovalue=0.008).

*SCXRD study*. The magnesium salt **Mg3d** containing the radical anion **3d^⋅–^** crystallized in the triclinic space group *P* $\bar{1}$ with *Z*=2 and two different complex cations as a double salt. The formula unit consists of the anion C_44_H_44_N_2_P_2_^−^ and 0.912 [Mg_2_Cl_3_(C_4_H_8_O)_6_]^+^ (cation **I** in Scheme 5, Figure 8 top) and 0.088 [MgCl(C_4_H_8_O)_5_]^+^ **⋅** (C_4_H_8_O) (cation **II** in Scheme 5, Figure S12) as well as one benzene molecule. These cationic, mono‐ and dinuclear magnesium chloride complexes[^49^, ^50^, ^51^, ^52^] and the equilibrium[^53^, ^54^] between them are well established in the literature and have recently been tested as Mg‐based battery electrolytes.[^55^, ^56^, ^57^] The cation [Mg_2_Cl_3_(C_4_H_8_O)_6_]^+^ has also been observed in the investigation of the constitution of Grignard reagents in THF by the means of X‐ray crystallography.^[58]^

There are no significant anion⋅⋅⋅cation interactions in the solid state for **Mg3d**. The molecular structure including the structural parameters within the P‐substituted indenyl ring (Table 2) of the radical anion **3d^⋅–^** is very similar to that of **2d** apart from the C−C bond lengths in the 1,2‐acenaphtyl backbone. Compared to **2d**, the C−C bond lengths in the 1,2‐acenaphtenyl substituent are significantly different, indicating that the unpaired electron is not located in the P‐substituted heterocycle but in the 1,2‐acenaphtenyl substituent of the imidazolyl backbone in accord with the computed spin density distribution (Figure 8 bottom, vide infra). Both the imidazolyl and the P‐substituted indenyl heterocycles are planar and linked by a slightly shortened central C−C single bond (*d*(C1−C2)=1.461(3) Å, Σ*r*_cov._(C−C) = 1.50 and Σ*r*_cov._(C=C) = 1.34 Å)^[33]^ displaying a small amount of double bond character. The P−C−C−N dihedral angle amounts to 60°, but the rotational potential around the central C−C axis can be assumed to be also very flat for this radical anion (vide supra), so that solid‐state effects during the crystallization process determine this value.

**Figure 9:**
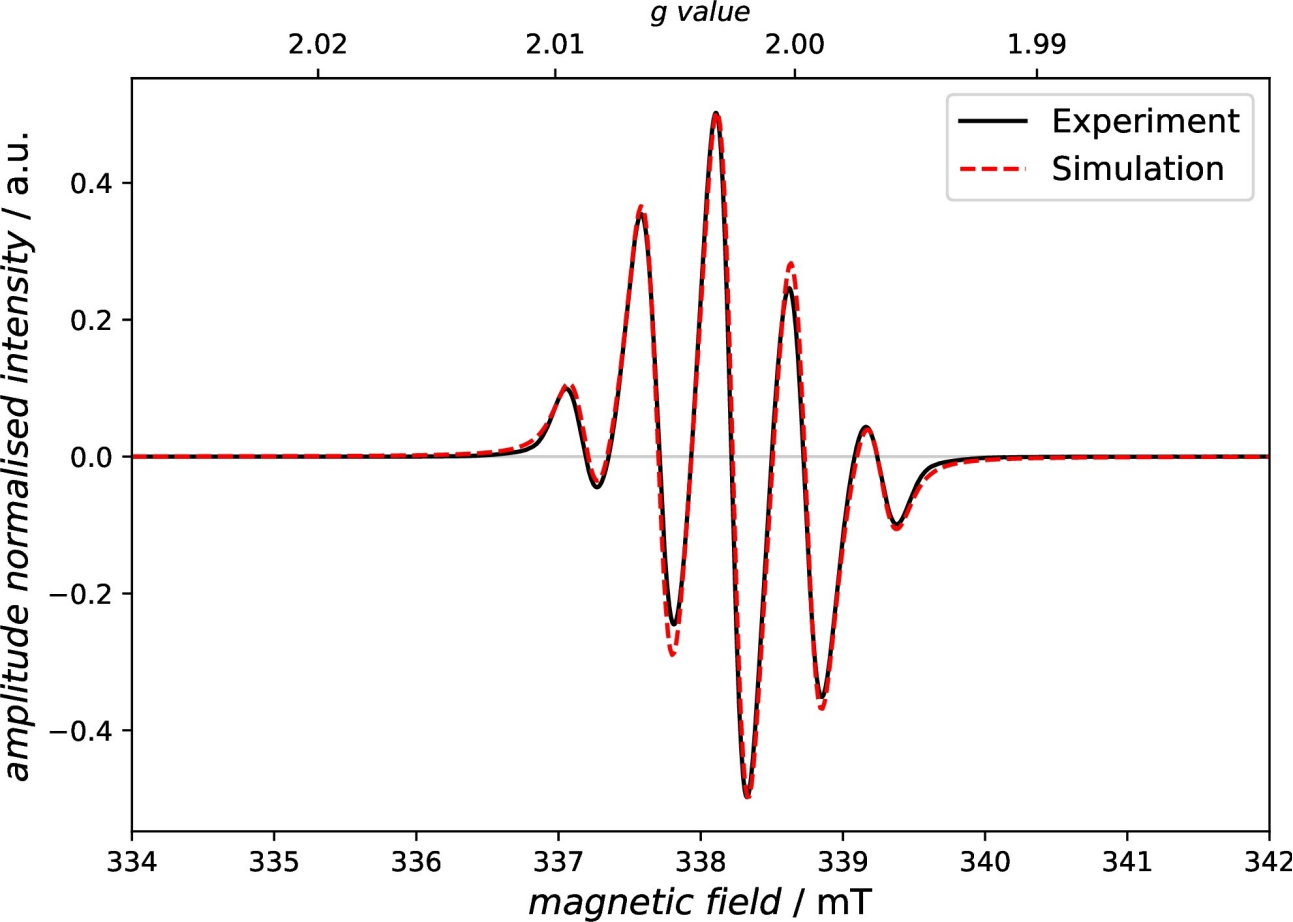
X‐band EPR spectra of **3d^⋅–^** at 298 K (Spectrum at 293 K see ESI).

*Electronic structure*. To prove experimentally the existence of the radical anion **3d^⋅^** ^–^ in solution, an X‐band EPR spectrum was measured at 298 K (2.7 mM in THF, Figure 9). The five‐line signal arises from coupling with four protons (H_A_, H_A’_, H_B_, H_B’_; Figure 8 bottom) of the 1,2‐acenaphtenyl substituent. Spectral simulation (see ESI) allowed the determination of isotropic hyperfine coupling to these four protons (*A*_iso_(^1^H_A_) = *A*_iso_(^1^H_A’_) = *A*_iso_(^1^H_B_)=*A*_iso_(^1^H_B’_)=14.6 MHz, with *g*_iso_=2.0026). As depicted in Figure 8 (bottom), the calculated spin density distribution shows that a 1,2‐acenaphthyl‐centred radical is present, where the spin density is mainly localized at four C atoms (C_A_ ‐ C_B’_).

**Figure 10:**
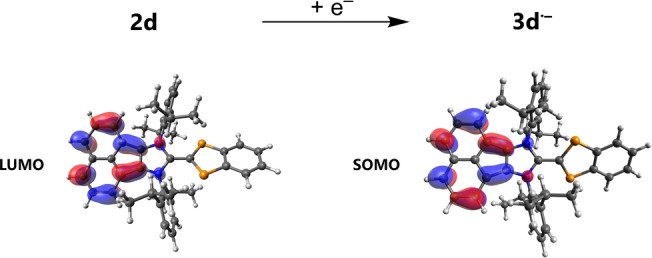
Calculated lowest unoccupied molecular orbital (LUMO) of **2d** (left) and singly occupied molecular orbital (SOMO) of **3d^⋅–^** (right, PBE0‐D3/def2‐TZVP(D) single point calculation, isovalue=0.04).

The spin density distribution can be explained on the basis of the LUMO in **2d**, which is exclusively delocalized over the 1,2‐acenaphthyl‐substituted imidazole ring and, in contrast to the LUMO in **2a**‐**2 c** (Figure 10, cf. Figure 6), there is no contribution from the P‐substituted indenyl heterocycle. This means that the reduction of **2d** (over‐reduction of **1d**), which is always observed during the preparation process of **2d** (Scheme 5), converts the LUMO of **2d** into the SOMO of **3d^⋅–^** by receiving an electron.

The overall bonding situation within the P‐substituted indenyl heterocycle is very similar to that in **2d**, i.e. the resonance between the Lewis structures **I**, **II** and **IV** in Scheme 6 is the best description in the Lewis picture, but with a monoradical 1,2‐acenaphthyl backbone.

### Activation of Small Molecules

**Scheme 7:**
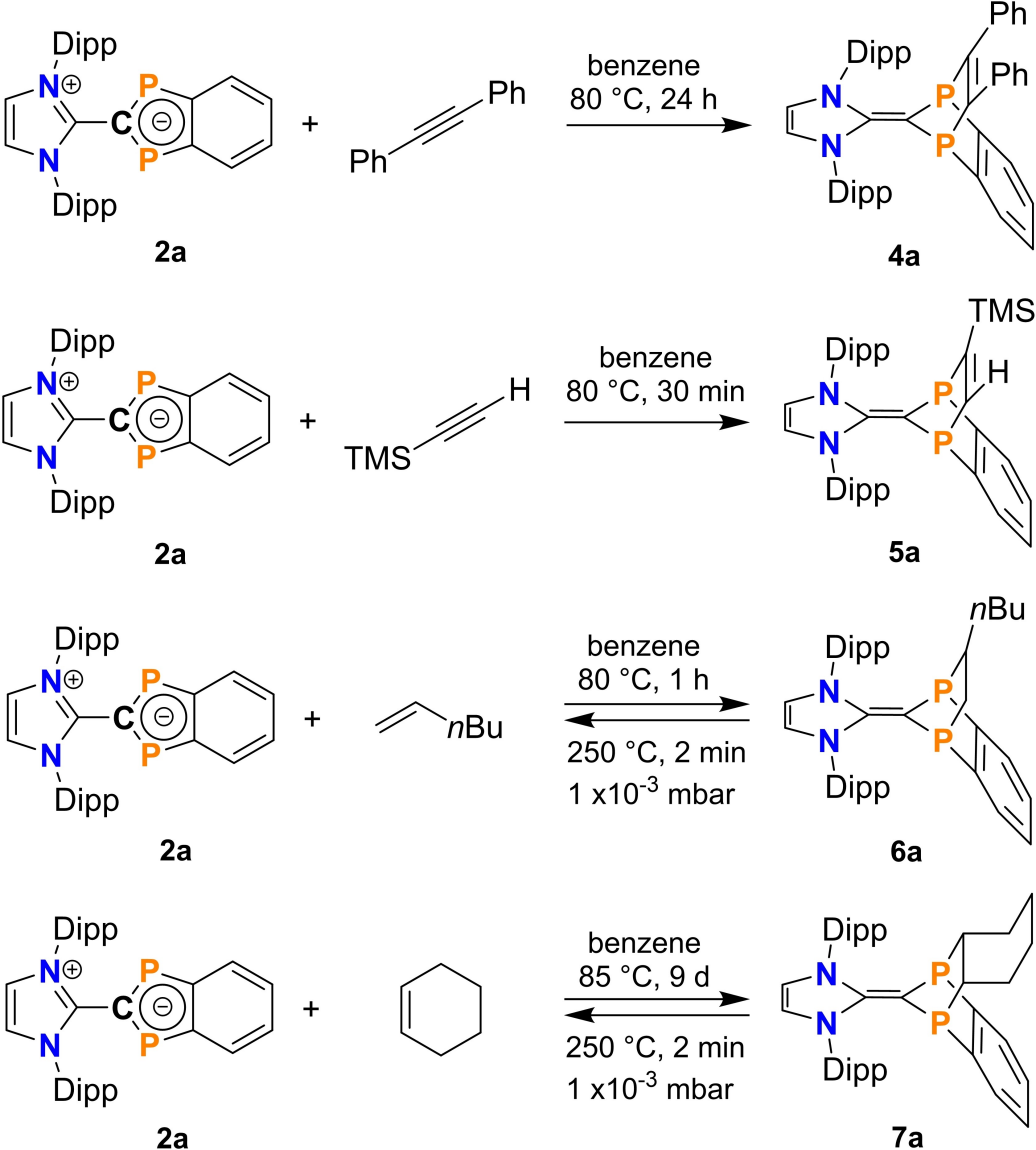
Reaction of **2a** with different alkynes and alkenes. The lower two alkene additions are fully reversible upon heating. Depicted Lewis formulae of **4a**‐**7a** represent the energetically most favored representation according to NBO analyses.

*General remarks*. The unusual bonding situation in diphospha‐indenylides of type **2** prompted us to investigate the extent to which species **2** are suitable for the activation of small molecules. To this end, two alkynes (Ph−C≡C−Ph, Me_3_Si−C≡CH) and two alkenes (*cyclo*‐hexene, hex‐1‐ene, Scheme 7) were analysed in detail in the reaction with **2**. The reaction with diphenylacetylene was carried out for all four species (**2a‐d**) and all other reactions only with **2a**.

**Figure 11:**
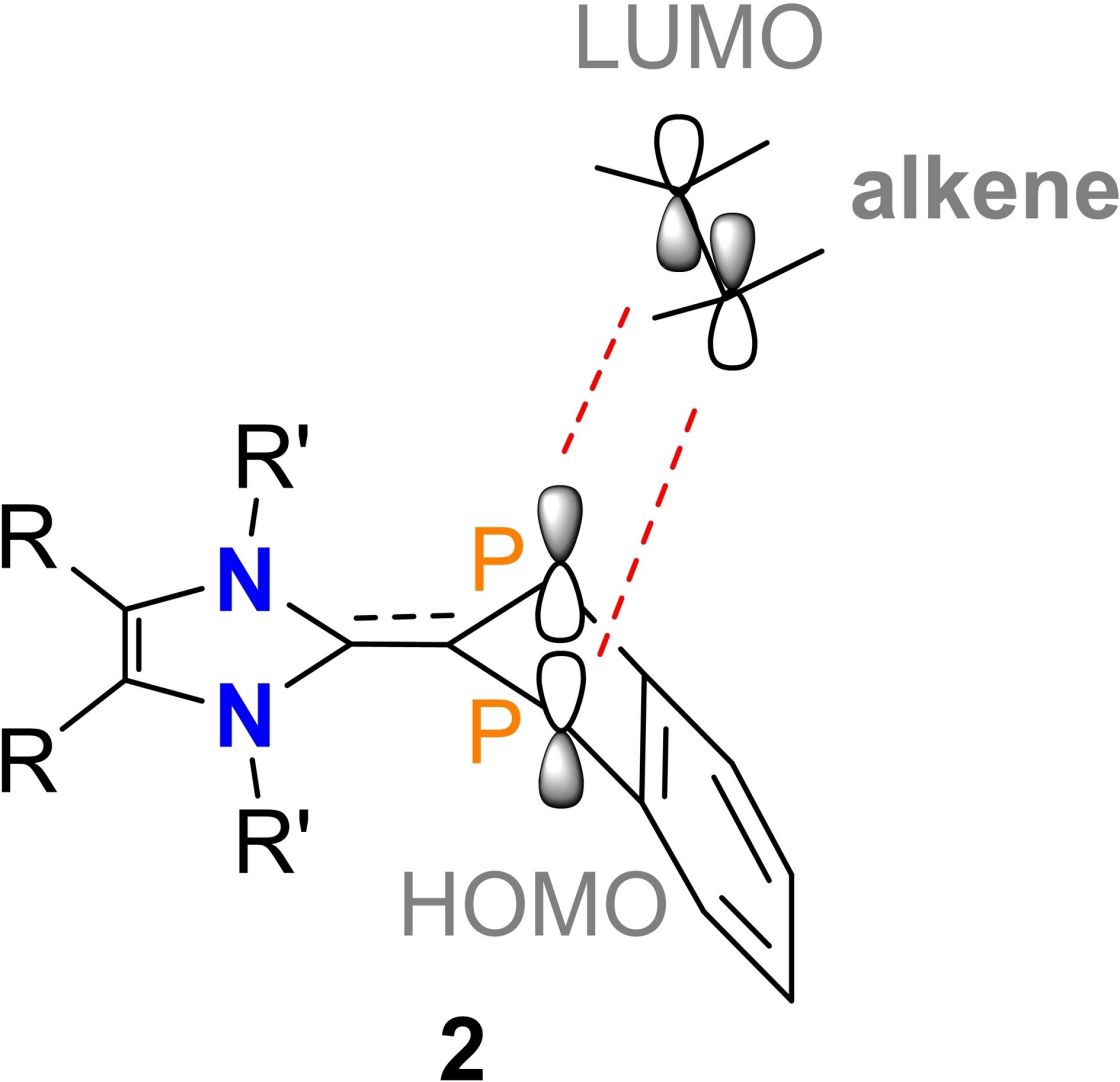
Schematic representation of the HOMO–LUMO interaction between **2** and an alkene.

For reasons of symmetry and from an electronic point of view, these formal addition reactions should work, since the HOMO of **2** (Figure 6) is trans‐annularly antibonding along the P−P axis, so that it can interact with the LUMO of the alkyne or alkene, which is also antibonding (cf. alkene C−C π*‐orbital, Figure 6 and Figure 11). The formal donation of electron density from the HOMO (**2**) to the LUMO (alkyne, alkenes) weakens the C−C bond and finally forms a [2.2.1] bicyclic cage compound in the sense of a pericyclic [2+2] cycloaddition.^[59]^ For steric reasons, however, this addition reaction can only take place when the indenyl heterocycle in **2** is co‐planar to the imidazolyl ring, which is possible since rotation around the central C−C bond is not hindered at ambient temperatures (vide infra)

The activation products **4a‐d**, **5a**, and **6‐7a** (Figure 6) are the first crystallographically verified examples of diphospha‐benzonorbornadiene and diphospha‐benzonorbornene derivatives, respectively. More importantly, the addition reactions have been shown to be reversible in some cases, an impetus for future applications in catalysis.

*Synthesis details*. All addition reactions were carried out in benzene at temperatures between 80 ‐ 90 °C in a closed vessel under autogenous pressure. During the reaction, compounds **2** dissolved in benzene and turned red in solution. The reaction can be traced by color change or ^31^P NMR spectroscopy.

*Addition of alkynes*. The reaction of **2a‐d** with diphenylacetylene led to the addition products **4a‐d** in good yields (69 ‐ 71 % wrt. isolated substance, Scheme 7). While compounds **4a‐c** were isolated as orange crystals, crystals of **4d** were deep blue (appearing black), similar in color to ^ANaph^IDipp=CH_2_. This indicates the presence of a C1−C2 double bond in these cases. Therefore, it can be concluded that the cages are disubstituted *N*‐heterocyclic olefines. The complete conversion of **2** to **4** was followed by ^31^P NMR experiments, as a significant up‐field‐shift occurs upon addition of the alkyne [cf. δ[^31^P]=190 ‐ 201 (**2**), 45 ‐ 49 ppm (**4**)]. All species were fully characterized including SCXRD (Figures S13 ‐ S16). The addition products **4** are all stable to moisture and air and have relatively high melting points (**4a**: 208, **4b**: 194, **4c**: 247, and **4d**: 295 °C). After we had shown that all four diphospha‐indenylides (**2**) show the same reactivity towards diphenylacetylene, we only used **2a** in the following experiments. Analogous to the reaction with diphenylacetylene, the asymmetrically substituted alkyne Me_3_Si−C≡CH also reacts with **2a**, even much faster than diphenylacetylene affording **5a** (Scheme 7). The reaction is completed within 30 min (yield 72 %). The ^31^P{^1^H} NMR spectrum of **5a** reveals that the two doublet signals are shifted upfield (13 and 7 ppm) with a ^2^ *J*(^31^P, ^31^P) coupling constant of 32 Hz. Compound **5a** starts to melt at 155°, but changes color at 202 °C in the melt from yellow to red hinting at a decomposition reaction (see Section on reversibility).

*Addition of alkenes*. Both alkenes used also add cleanly to **2a** at 80 °C in benzene, but while hex‐1‐ene quickly adds to **2a** within one hour, the reaction with *cyclo*‐hexene takes nine days to complete (Scheme 7). The hex‐1‐ene addition product **6a** (δ[^31^P]=8.2, 7.2 ppm as doublets) and the *cyclo‐*hexene product **7a** (δ[^31^P]=18.8 ppm) were isolated as colorless needles with a yield of 68 % and 47 %, respectively. When heated both colorless compounds start to turn red before melting (Mp: **6a**: 116, **7a**: 287 °C). In case of **7a** the melting point is equal to the melting point of the starting material **2a**. This color change observation prompted us to investigate the release of the alkenes or alkynes, i.e. the reversibility of the addition reaction, in more detail experimentally (next section). Since **4a‐d** are stable in the melt without any observable color changes (remaining yellow or blue, see ESI), they were not included in these investigations.

*Reversibility*. To investigate reversibility of the alkene/alkyne addition, a series of different experiments was carried out in which temperature, heating time and pressure were varied (see ESI section 6.3). The decomposition products **2a** and alkene/alkyne were detected by NMR (**2a**) or IR spectroscopy in the gas phase (alkene/alkyne). It was found that when **5a**, **6a**, and **7a** were heated in the solid state to 150 °C for 5 minutes in static vacuum, they partly decomposed, and the corresponding alkene/alkyne could be observed in the gas phase. However, as this reaction did not proceed quantitatively under these conditions, the reaction conditions were changed. A quantitative reverse reaction for **6a** and **7a** was achieved at 250 °C in a heated aluminium block under dynamic vacuum conditions for 2 minutes. For **5a**, no conditions were found that allowed a complete reverse reaction without further decomposition to unknown products. Furthermore, it was impossible to reverse the addition reaction for compound **4a** without triggering undesirable decomposition.

**Figure 12:**
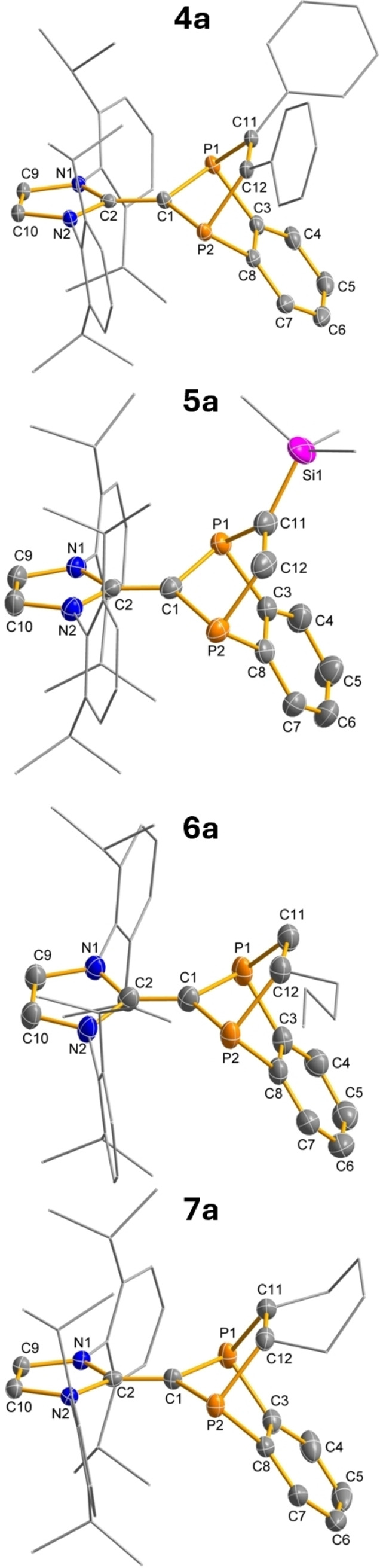
Molecular structures of **4a**, **5a**, **6a** and **7a** in the crystal (*T*=123(2) K, ellipsoids at 50 % probability). R’ and olefin substituent shown as wireframe. H atoms omitted for clarity. Selected bond lengths and angles are listed in Table 2.

*Structure and bonding*. All addition products were studied by SCXRD. The molecular structures of **4a**‐**7a** in the crystal are depicted in Figure 12. Probably the most prominent change in the molecular structure after addition of the alkene/alkyne concerns the P‐substituted indenyl ring, which is now considerably folded along the P−P axis, while the imidazolyl ring remains planar. In addition, the central C−C distance (between 1.376 ‐ 1.392, cf. 1.455(2) Å in **2a**, Table 2) is significantly shorter and is now in the region of a typical C−C double bond (cf. Σ*r*_cov._(C−C) = 1.50 and Σ*r*_cov._(C=C) = 1.34 Å).^[33]^ Moreover, the P−C bonds are elongated and the P−C−P angles smaller due to the increased steric strain upon cage formation. The C−C bonds of the former alkyne/alkene are also elongated and lie in the region of a single bond (**6a**: 1.542(5), **7a**: 1.557(2) Å) or double bond (**4a**: 1.347(2), **5a**: 1.331(5) Å). In accordance with these structural data, the NBO analysis for all addition products shows a significant reduction of the charge transfer between the imidazolyl ring and the *P*‐heterocycle [0.403 ‐ 0.436e (**4a**‐**7a**); cf. 0.737e (**2**)]. Quantum chemical calculations show that all addition products are closed‐shell singlet species.

## Conclusion

In summary, the linkage of NHOs with a P‐substituted indenyl heterocycle was achieved in good yield. These NHO‐linked *P*‐indenylides (**2**) are zwitterionic molecules with a small amount of open‐shell singlet diradical character. Quantum chemical calculations show that the central C−C bond between the imidazolyl and the phosphorus‐substituted indenyl ring corresponds to a single bond with a slight double bond character, so that rotation around this bond is thermally possible at ambient temperatures. This rotation in turn causes a change in the diradical character depending on the dihedral angle between the two heterocycles and thus also the HOMO–LUMO gap, which makes these compounds thermochromic.

Furthermore, it was possible to study the intricate interplay between zwitterionic, diradical, and aromatic character, which depend on the above‐mentioned rotation around the C−C bond. In particular, the aromaticity of **2** rises with increasing dihedral angle between the ring planes, while the diradical character decreases.

Different substitution on the NHO plays only a minor role in influencing the electronic structure, unless an 1,2‐acenaphthyl substituent is incorporated into the backbone of the NHO instead of alkyl substituents. Again, there is an additional distinct delocalized π‐electron system in the acenaphthyl that alters the HOMO–LUMO gap of the entire molecule as well as the nature of the LUMO. While in the case of the alkyl‐substituted NHO linked *P*‐indenylides, the LUMO is mainly localized in the indenyl ring, in the case of the acenaphthyl‐substituted species the LUMO is mainly localized in the NHO. This in turn means that no thermochromism is observed for this species and this acenaphtyl moiety is also easily reduced. The synthesis of acenaphtyl‐substituted *P*‐indenylides therefore always produces a magnesium salt as side‐product that contains a radical anion, which could be isolated and fully characterized.

With the help of the NHO‐linked *P*‐indenylides, alkenes and alkynes can be easily activated, which leads to the formation of [2.2.1] bicyclic cage compounds. The latter are neither zwitter ionic nor diradicaloid but closed‐shell singlet species. The addition reactions of the alkenes are completely reversible, while the alkyne adducts only partially thermally dissociate before complete decomposition sets in.

This new class of NHO‐linked *P*‐indenylides with their intriguing electronic properties will allow further reactivity studies with the aim of investigating the suitability of **2** for metal‐free homogeneous catalysis.

## Experimental Section

Experimental section, preparation of starting materials and compounds, structure elucidation, additional spectroscopic details and computational details can be found in the Supporting Information.

Computations were carried out using Gaussian09^[60]^, ORCA 4.2.1^[61]^ or ORCA 5.0.3^[62]^ and the standalone version of NBO 6.0.[^36^, ^63^, ^64^, ^65^]

## Conflict of Interests

The authors declare no conflict of interest.

1

## Supporting information

As a service to our authors and readers, this journal provides supporting information supplied by the authors. Such materials are peer reviewed and may be re‐organized for online delivery, but are not copy‐edited or typeset. Technical support issues arising from supporting information (other than missing files) should be addressed to the authors.

Supporting Information

## Data Availability

The data that support the findings of this study are available in the supplementary material of this article.
