# Supplementary material for: Reaction of NHOs with Bisphosphanes – Designing Diradicaloids, Zwitterions and Radicals
Source: Angew Chem Int Ed Engl. 2025 Feb 13;64(15):e202423347. doi: 10.1002/anie.202423347 (PMC11976208; doi:10.1002/anie.202423347)
Supplement: Supplementary file 1 — Supporting Information [file ANIE-64-e202423347-s001.pdf]

Angewandte Chemie
Eine Zeitschrift der Gesellschaft Deutscher Chemiker

Supporting Information

Reaction of NHOs with Bisphosphanes – Designing Diradicaloids, Zwitterions and Radicals

K. P. Lüdtke, E. Zander, F. Taube, J.-E. Siewert, B. Corzilius, C. Hering-Junghans, J. Bresien, A. Schulz**

Supporting Information

High Pressure Synthesis of Pr_2O_5 – A Unique Lanthanoid(IV) Oxide Peroxide

N. T. Flosbach[1], L. Brüning[2], P. L. Jurzick[1,2], E. Bykova[3], M. Ekholm[4], I. A. Abrikosov[4], M. Amirabbasi[5], M. Mezouar[6], B. Wehinger[6], N. Giordano[7], T. Fedotenko[7], V. Prakapenka[8], S. Chariton[8], M. S. Wickleder,[1] M. Bykov[2]

[1]University of Cologne, Institute of Inorganic Chemistry, Greinstr. 6, 50939 Cologne, Germany

[2]Institute of Inorganic and Analytical Chemistry, Goethe University Frankfurt, Max-von-Laue-Str. 7, 60438 Frankfurt am Main, Germany

[3]Institute of Geosciences, Goethe University Frankfurt, Altenhöferallee 1, 60438 Frankfurt am Main, Germany

[4]Department of Physics, Chemistry and Biology, Linköping University, SE-581 83, Sweden

[5] Fachgebiet Materialmodellierung, Institut für Materialwissenschaft, Technische Universität Darmstadt, Otto-Berndt-Str. 3, D-64287 Darmstadt, Germany

[6]European Synchrotron Radiation Facility, Grenoble Cedex F-38043, France

[7]Deutsches Elektronen-Synchrotron DESY, Notkestr. 85, 22607 Hamburg, Germany

[8]Center for Advanced Radiation Sources, University of Chicago, Chicago, IL, USA

Table of Contents

Experimental and Computational Details 2
Synthesis of Praseodymium Dioxide 2
Reaction of Praseodymium Dioxide and Oxygen 2
Control Experiment 3
X-Ray Diffraction Data Collection and Processing 3
Quantum Theoretical Calculations 3
Crystallographic Data 4
Raman Spectrum 6
XRD Maps 6

Experimental and Computational Details

Synthesis of Praseodymium Dioxide

250 mg of Pr_6O_{11} (obtained from calcination of freshly precipitated $Pr(OH)_3$) (0.245 mmol, 6 eq.) were added to a silica crucible that was inserted into a steel autoclave with an internal volume of ca. 20 ml. 9 ml of liquid oxygen were condensed into the autoclave before it was closed and heated to 500 °C for 12 hours. After allowing the autoclave to cool to room temperature, the pressure was released and 254 mg of PrO_2 (1.47 mmol, 100%) were retrieved from the silica crucible as a black powder.

Reaction of Praseodymium Dioxide and Oxygen

Praseodymium dioxide was loaded in a BX90-type diamond anvil cell equipped with Boehler-Almax-type diamonds (culet diameter 250 μm) together with oxygen under cryogenic loading. Oxygen served both as a pressure transmitting medium and a reactant. The sample was compressed to 27 GPa and laser heated to ca. 1400 °C. The structure was solved by single crystal X-ray diffraction of multi grain samples at beam line P02.2 at DESY. The pressure was then reduced to 20 GPa, 11 GPa and finally to ambient pressure without further heating. At both 20 GPa and 11 GPa, the title compound could be identified by single crystal diffraction data. At ambient pressure, the diffraction patterns did not allow for satisfactory structure solution. Raman data of the cell was recorded at 27 GPa using the advanced integrated optical spectroscopy system at GSECARS.[1]

Control Experiment

Praseodymium dioxide was loaded in a BX90 diamond anvil cell equipped with Boehler-Almax-type diamonds (culet diameter 250 μm) together with argon. The cell was compressed to 33 GPa and heated to >1500 K. The cell was decompressed to first 28 GPa and then to ambient pressure. At all three pressure points single crystal diffraction data was collected. The cotunnite-type PrO_2 phase was identified at 33 GPa and 28 GPa, whereas no satisfactory structure solutions could be obtained at ambient pressure.[2]

X-Ray Diffraction Data Collection and Processing

X-Ray diffraction data was collected at the synchrotron beamline P02.2 at DESY (λ = 0.2903 Å, Perkin Elmer XRD1621 flat panel detector).[3-4] The control experiment was conducted at the synchrotron beam line ID27 at ESRF (λ = 0.3738 Å, Eiger2 X 9M CdTe flat panel detector).[5] After increasing the cell pressure to the target pressure (pressure determined by Raman shift of the diamond D-band),[6] a two dimensional grid of the heated area was produced by collecting XRD images at the respective positions. ω-scans of ±35° were collected at selected positions within the grid. The diffraction data was processed and analysed with the CrysAlisPro software in combination with DaFi.[7] Structures were solved and refined using ShelX with the Olex user interface.[8-9] The full data sets were deposited in the Cambridge crystal structure database (CCDC) with the numbers 2303283 (27 GPa), 2303282 (20 GPa) and 2303284 (11 GPa).

Quantum Theoretical Calculations

Ab-initio density functional theory (DFT)[10-11] calculations were performed with the projector augmented waves (PAW) method[12-13] as implemented in the Vienna ab-initio simulation package (VASP) [14-16]. We used the revised Perdew-Burke-Ernzerhof generalized gradient approximation (PBEsol)[17] with the on-site effective Coulomb parameter U_{eff} = 2.5 eV in the parametrization by Dudarev et al. [18-19]. Structural relaxation was performed with the plane wave expansion up to 525 eV and an 8x6x8 k-point grid. Total energy was converged within 10^{-6} eV and the forces were converged within 0.01 eV / Å. Presented calculations were done for an antiferromagnetically ordered cell within the scalar-relativistic approximation. Test calculations show that the magnetic configuration and spin-orbit coupling only have a very minor impact on the quantities of interest.

Crystallographic Data

Tab. S 1: Selected crystallographic data of $Pr_2(O_2)O_3$ at three different pressure points.

	**11 GPa**	**20 GPa**	**27 GPa**
Chemical Formula	Pr_2O_5	Pr_2O_5	Pr_2O_5
Molar Mass	361.82 g/mol	361.82 g/mol	361.82 g/mol
Temperature	293(2) K	293(2) K	293(2) K
Crystal System	orthorhombic	orthorhombic	orthorhombic
Space Group	*Pbcn*	*Pbcn*	*Pbcn*
Lattice Parameters	a = 617.2(1) pm<br>b = 802.7(1) pm<br>c = 616.33(8) pm	a = 606.1(1) pm<br>b = 794.2(1) pm<br>c = 611.7(2) pm	a = 601.4(2) pm<br>b = 779.6(2) pm<br>c = 610.5(1) pm
Cell Volume	$305.33(3)\cdot10^6$ pm^3	$294.4(1)\cdot10^6$ pm^3	$286.19(1)\cdot10^6$ pm^3
Formula Units	4	4	4
Calculated Density	7.871 g/cm^3	8.162 g/cm^3	8.397 g/cm^3
Absorption Correction	multi scan	multi scan	multi scan
Absorption Coefficient	15.035 mm^{-1}	15.592 mm^{-1}	16.040 mm^{-1}
F(000)	632.0	632.0	632.0
Crystal Size	0.001 × 0.001 × 0.001 mm^3	0.001 × 0.001 × 0.001 mm^3	0.001 × 0.001 × 0.001 mm^3
Radiation	synchrotron (λ = 0.2901)	synchrotron (λ = 0.2901)	synchrotron (λ = 0.2901)
2θ-Range	3.398 to 35.17°	3.45 to 35.544°	3.492 to 34.54°
Index Range	$-8 \le h \le 8, -12 \le k \le 13, -9 \le l \le 10$	$-10 \le h \le 9, -15 \le k \le 14, -9 \le l \le 9$	$-8 \le h \le 8, -11 \le k \le 13, -10 \le l \le 9$
Reflections Collected	1472	1530	1447
Independent Reflections	708 [R_{int} = 0.0150, R_σ = 0.0178]	667 [R_{int} = 0.0179, R_σ = 0.0236]	642 [R_{int} = 0.0083, R_σ = 0.0090]
Data/Restraints/Parameters	708/0/33	667/0/33	642/0/34
Goodness of Fit	1.087	1.283	1.125
R_1; wR_2 ($I\geq2\sigma(I_o)$)	R_1 = 0.0388, wR_2 = 0.1094	R_1 = 0.0361, wR_2 = 0.0901	R_1 = 0.0252, wR_2 = 0.0676
R_1; wR_2 (All Data)	R_1 = 0.0488, wR_2 = 0.1173	R_1 = 0.0486, wR_2 = 0.0940	R_1 = 0.0277, wR_2 = 0.0685
Largest Diff. Peak/Hole	7.10/-2.78	2.65/-2.98	2.34/-2.75

Tab. S 2: Summary of selected interatomic distances as well as other structural parameters of $Pr_2(O_2)O_3$ at different pressures.

	**11 GPa**	**20 GPa**	**27 GPa**
Cell Volume / 10^6 pm^3	305.33(3)	294.4(1)	286.19(1)
a / pm	617.2(1)	606.1(1)	601.4(2)
b / pm	802.7(1)	794.2(1)	779.6(2)
c / pm	616.33(8)	611.7(2)	610.5(1)
d(O1-O1) / pm	147(1)	145(1)	144.1(7)
$V_{Polyhedron}$ [PrO_{10}] / 10^6 pm^3	285.09(6)	275.03(7)	267.29(2)
d_{avg}(Pr-O) / pm	238.4(5)	235.5(6)	233.3(4)
d_1(Pr1-O1) / pm	240.2(5)	238.5(6)	235.5(4)
d_1(Pr1-O2) / pm	233.0(5)	231.4(7)	228.0(4)
d_2(Pr1-O2) / pm	232.1(5)	228.7(6)	229.7(4)
d_1(Pr1-O3) / pm	237.7(3)	234.3(3)	230.6(2)
d_3(Pr1-O2) / pm	221.4(5)	219.1(6)	235.2(4)
d_4(Pr1-O2) / pm	234.7(5)	231.8(6)	228.1(4)
d_2(Pr1-O1) / pm	256.0(5)	253.5(7)	245.3(4)
d_3(Pr1-O1) / pm	242.3(5)	238.5(6)	265(4)
d_2(Pr1-O3) / pm	234.5(3)	231.9(3)	232.4(2)
d_4(Pr1-O1) / pm	252.2(5)	247.4(6)	252.0(4)

Raman Spectrum

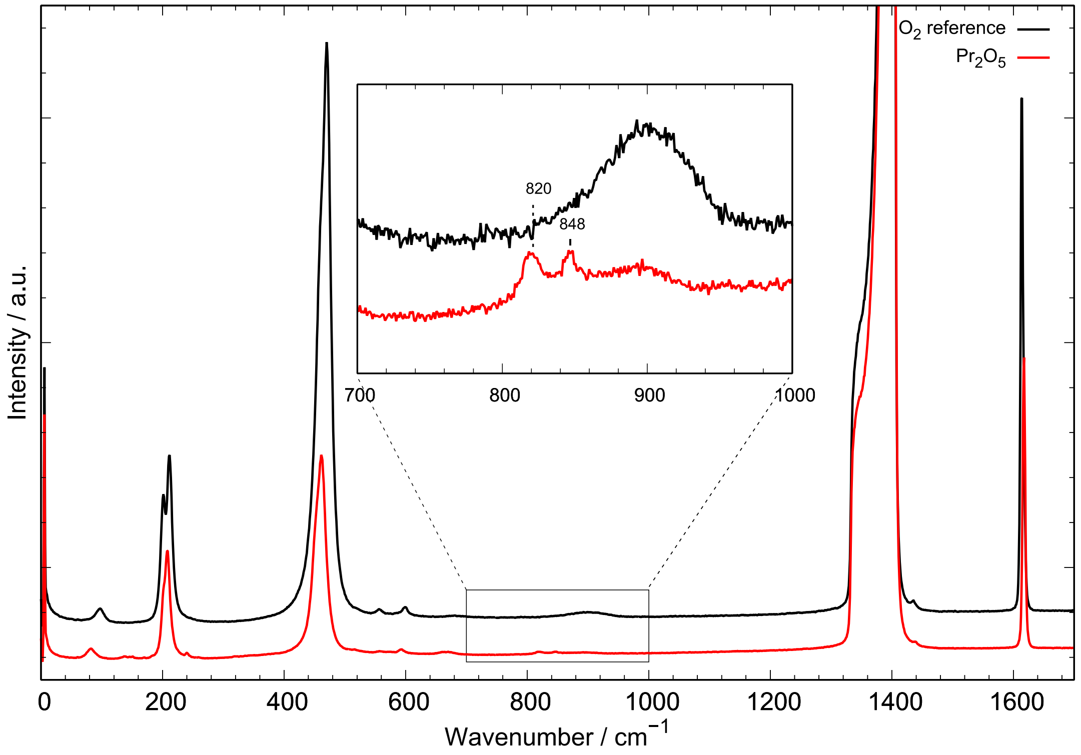

Fig. S 1: Raman spectrum of the reaction chamber at 27 GPa. Vibrations assigned to the peroxide group of $Pr_2(O_2)O_3$ are highlighted. The spectra were recorded with an excitation wavelength of 532 nm. The black spectrum is a reference spectrum of only oxygen without praseodymium oxides that was measured using the same experimental setup.

XRD Maps

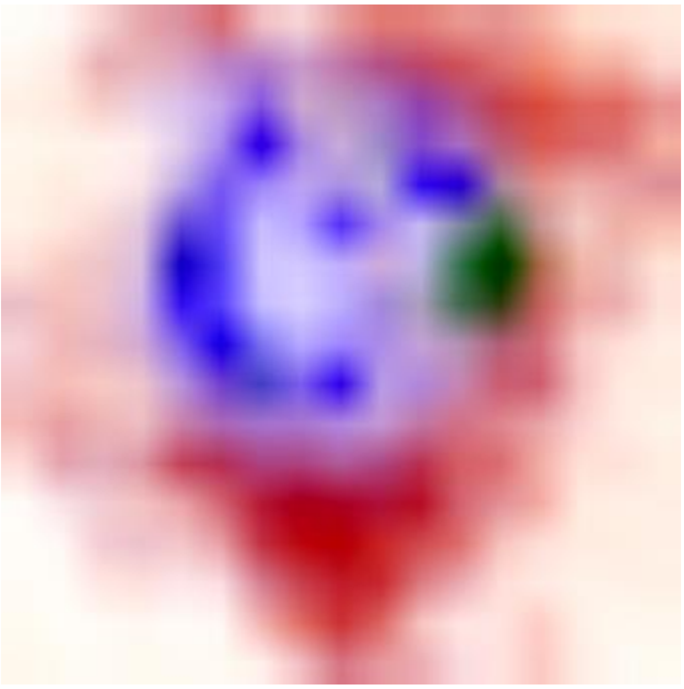

Fig. S 2: X-Ray diffraction map of the heated area in the diamond anvil cell at 27 GPa. Each edge of the graphic corresponds to 300 µm in the actual cell. Red = fluorite PrO_2; blue = $Pr_2(O_2)O_3$; green = ruby.

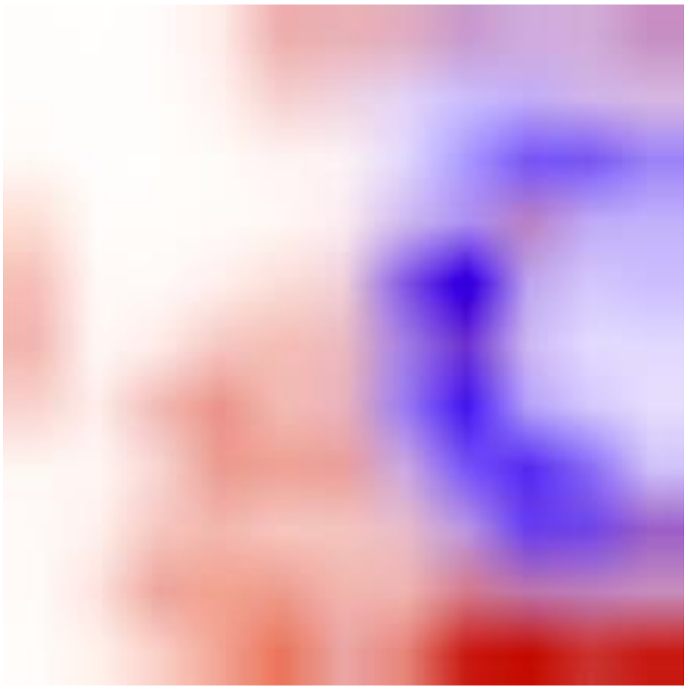

Fig. S 3: X-Ray diffraction map of the heated area in the diamond anvil cell at 20 GPa. Each edge of the graphic corresponds to 220 μm in the actual cell. Red = fluorite PrO_2; blue = $Pr_2(O_2)O_3$.

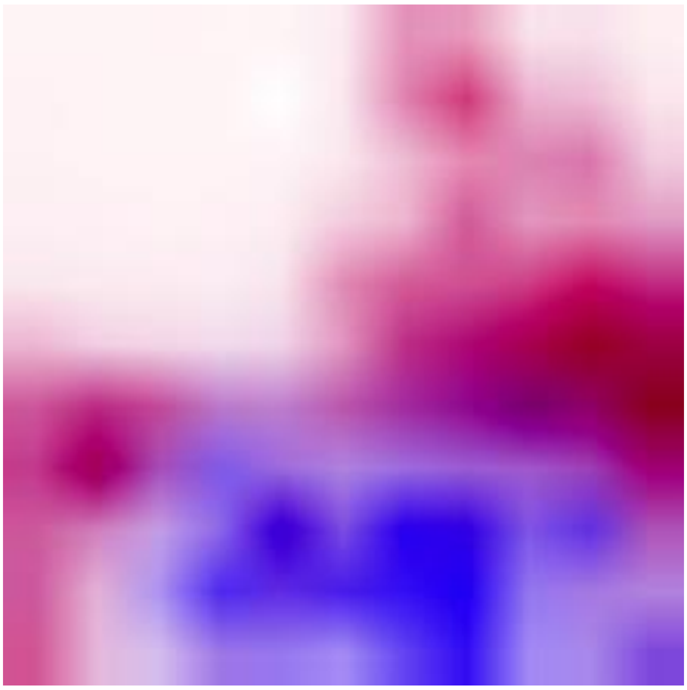

Fig. S 4: X-Ray diffraction map of the heated area in the diamond anvil cell at 11 GPa. Each edge of the graphic corresponds to 220 μm in the actual cell. Red = fluorite PrO_2; blue = $Pr_2(O_2)O_3$.

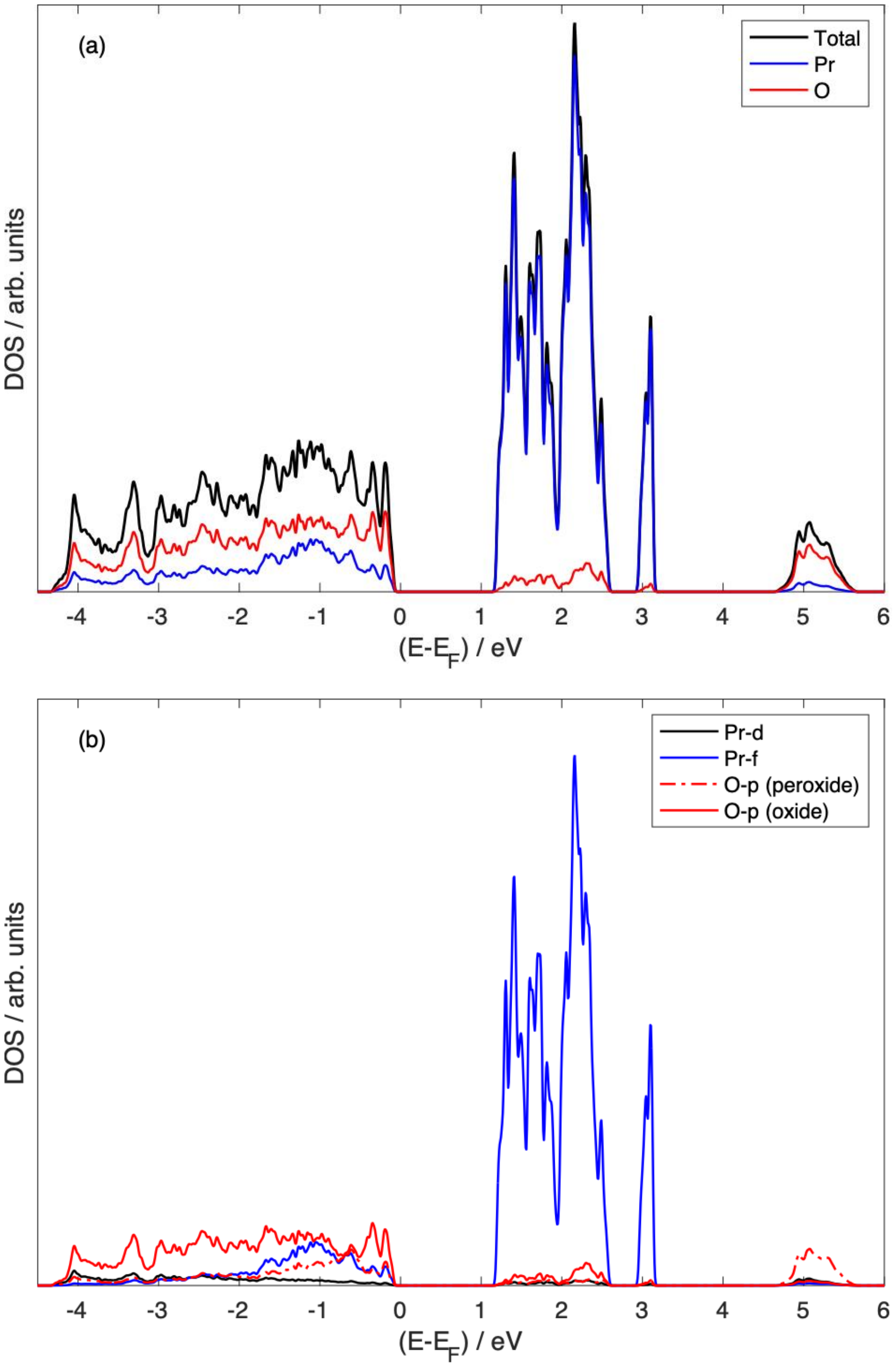

Fig. S 5: Calculated density of states (DOS) at the volume 290 Å^3. (a): total and site-projected DOS with respect to the Fermi energy. (b): orbital resolved DOS showing Pr *d*- and *f*-states and *p*-states of oxide and peroxide anions. Note the antibonding peroxide states at E-E$_F$ = 5 eV. The peak contains exactly 2 electron states per formula unit.

References

[1] N. Holtgrewe, E. Greenberg, C. Prescher, V. B. Prakapenka, A. F. Goncharov, *High Press. Res.* **2019**, *39*, 457-470.

[2] A. Haberer, H. Huppertz, *Z. Anorg. Allg. Chem.* **2010**, *636*, 363-367.

[3] E. Bykova, G. Aprilis, M. Bykov, K. Glazyrin, M. Wendt, S. Wenz, H.-P. Liermann, J. T. Roeh, A. Ehnes, N. Dubrovinskaia, L. Dubrovinsky, *Rev. Sci. Instrum.* **2019**, *90*, 073907.

[4] H.-P. Liermann, Z. Konopkova, W. Morgenroth, K. Glazyrin, J. Bednarcik, E. E. McBride, S. Petitgirard, J. T. Delitz, M. Wendt, Y. Bican, A. Ehnes, I. Schwark, A. Rothkirch, M. Tischer, J. Heuer, H. Schulte-Schrepping, T. Kracht, H. Franz, *J. Synchrotron Rad.* **2015**, *22*, 908-924.

[5] S. Petitgirard, A. Salamat, P. Beck, G. Weck, P. Bouvier, *J. Synchrotron Rad.* **2014**, *21*, 89-96.

[6] Y. Akahama, H. Kawamura, *J. Appl. Phys.* **2006**, *100*, 043516.

[7] A. Aslandukov, M. Aslandukov, N. Dubrovinskaia, L. Dubrovinsky, *J. Appl. Crystallogr.* **2022**, *55*, 1383-1391.

[8] G. M. Sheldrick, *Acta Crystallogr. A* **2008**, *64*, 112-122.

[9] O. V. Dolomanov, L. J. Bourhis, R. J. Gildea, J. A. K. Howard, H. Puschmann, *J. Appl. Crystallogr.* **2009**, *42*, 339-341.

[10] P. Hohenberg, W. Kohn, *Phys. Rev.* **1964**, *136*, B864-B871.

[11] W. Kohn, L. J. Sham, *Phys. Rev.* **1965**, *140*, A1133-A1138.

[12] P. E. Blöchl, *Phys. Rev. B* **1994**, *50*, 17953-17979.

[13] G. Kresse, D. Joubert, *Phys. Rev. B* **1999**, *59*, 1758.

[14] G. Kresse, J. Hafner, *Phys. Rev. B* **1993**, *47*, 558.

[15] G. Kresse, J. Furthmüller, *Comput. Mater. Sci.* **1996**, *6*, 15-50.

[16] G. Kresse, J. Furthmüller, *Phys. Rev. B* **1996**, *54*, 11169.

[17] J. P. Perdew, A. Ruzsinszky, G. I. Csonka, O. A. Vydrov, G. E. Scuseria, L. A. Constantin, X. Zhou, K. Burke, *Phys. Rev. Lett.* **2008**, *100*, 136406.

[18] V. I. Anisimov, J. Zaanen, O. K. Andersen, *Phys. Rev. B* **1991**, *44*, 943.

[19] S. L. Dudarev, G. A. Botton, S. Y. Savrasov, C. J. Humphreys, A. P. Sutton, *Phys. Rev. B* **1998**, *57*, 1505.
